# Accounting for non-stationarity in epidemiology by embedding time-varying parameters in stochastic models

**DOI:** 10.1371/journal.pcbi.1006211

**Published:** 2018-08-15

**Authors:** Bernard Cazelles, Clara Champagne, Joseph Dureau

**Affiliations:** 1 Institut de Biologie de l’Ecole Normale Supérieure (IBENS), Ecole Normale Supérieure, CNRS UMR 8197, Paris, France; 2 International Center for Mathematical and Computational Modeling of Complex Systems (UMMISCO), UMI 209, UPMC/IRD, France; 3 Hosts, Vectors and Infectious Agents, CNRS URA 3012, Institut Pasteur, Paris, France; 4 CREST, ENSAE, Université Paris Saclay, Palaiseau, France; 5 SNIPS, Paris, France; Emory University, UNITED STATES

## Abstract

The spread of disease through human populations is complex. The characteristics of disease propagation evolve with time, as a result of a multitude of environmental and anthropic factors, this non-stationarity is a key factor in this huge complexity. In the absence of appropriate external data sources, to correctly describe the disease propagation, we explore a flexible approach, based on stochastic models for the disease dynamics, and on diffusion processes for the parameter dynamics. Using such a diffusion process has the advantage of not requiring a specific mathematical function for the parameter dynamics. Coupled with particle MCMC, this approach allows us to reconstruct the time evolution of some key parameters (average transmission rate for instance). Thus, by capturing the time-varying nature of the different mechanisms involved in disease propagation, the epidemic can be described. Firstly we demonstrate the efficiency of this methodology on a toy model, where the parameters and the observation process are known. Applied then to real datasets, our methodology is able, based solely on simple stochastic models, to reconstruct complex epidemics, such as flu or dengue, over long time periods. Hence we demonstrate that time-varying parameters can improve the accuracy of model performances, and we suggest that our methodology can be used as a first step towards a better understanding of a complex epidemic, in situation where data is limited and/or uncertain.

## Author summary

As our world becomes more and more globalized, infectious disease poses an ever-increasing threat to human health. The multitude of environmental and behavioral factors, which account for the spread of infectious diseases, are ever-evolving and thus infectious diseases propagation is complex.

In the face of this complexity, mathematical models offer valuable tools to study the dynamics of epidemic diseases. Developing adequate statistical and mathematical tools, that take account of the time-varying nature of the different mechanisms responsible for disease propagation, remains a major challenge. To take this increasingly important aspect into consideration, we propose a flexible methodology that encompasses time-varying aspects of the epidemic. It does this via diffusion process equations for time-varying parameters. Considering the relative paucity of available data, our principal assertion is that it is preferable to use this flexible framework with time-varying parameters, that tracks epidemiological patterns, and updates the key parameters according to data, than to use a more complex model.

## Introduction

Our world constantly faces the threat of emerging and re-emerging diseases and it has been shown that this has intensified over the past 50 years. This intensification is due, in part, to climate change, urbanization and globalization [1] meaning that infectious diseases remain a constant and unpredictable threat to human health.

Numerous factors contribute to the propagation of an infectious disease. These include increased human connectivity, limited availability of economic resources for adequate intervention, increasing antimicrobial resistance, evolution of the dominant strains and increasing parasite and vector resistance to the most widely used drugs and insecticides, etc. A key factor in this huge complexity is non-stationarity [2], meaning that the characteristics of the dynamical epidemiological processes evolve with time. Thus, the mechanisms of transmission are uncertain, making it difficult to obtain quantitative predictions. One of the classic aspects of non-stationarity, is the seasonality of epidemiological dynamics, linked to environment and climate [3–4] but the environmental variability can shape the disease propagation in unforeseeable ways on small and large spatial scales [5–8]. Intervention and control may also modify the course of an epidemic. A less well-described but equally important cause of non-stationarity is linked to social cycles, *e*.*g*. school terms, religious holidays and agricultural cycles [9–13]. Research increasingly focuses on the effect of behavioral change in the presence of epidemiological risk as a source of non-stationarity [14–16]. Societal responses and changing human behavior play an important role in our connected society. Thus, during an epidemic, depending on the availability of information on the disease, people exhibit a variety of behaviors including anxiety and social distancing that might greatly influence the course of an epidemic.

For all of the above reasons, the spread of pathogens through human populations can be complex and hard to predict. In the face of this complexity, mathematical models offer valuable tools to study the dynamics of epidemic diseases, in order to synthesize information to understand observed epidemiological patterns and to test different hypothesis on the underlying key mechanisms [17]. Moreover, mathematical models play a crucial role in infectious disease prevention by assessing the impact of different control measures, *e*.*g*. vaccination strategies [18–19].

Nonetheless, there are very few, if indeed any, cases where modelers can access all the necessary information to reliably predict the course of an epidemic. This is particularly the case when we consider the non-stationarity features of epidemics and their transient nature poses a challenging problem for modeling. Further to this, different hypothesis must be formulated. In the case of influenza, for example, some researchers have suggested using a quantitative relationship between climatic variables and the effective transmission rate [20]. Another recent example illustrates non-stationarity in epidemiology. Between November 2010 and February 2011, despite a low level of population susceptibility, an unexpected third wave of infection by the H1N1pdm09 pandemic virus was observed in the United Kingdom. Using a compartmental mathematical model of influenza transmission, this third wave was explained, by a substantial increase in the transmissibility of the H1N1pdm09 virus [21]. It has been proposed that this modification of the transmissibility was caused by the virus evolution with a better adaptation to the human host, or by climatic factors, namely the very cold weather experienced in the United Kingdom at that time, or by a combination of these factors [21].

To tackle the problem of non-stationarity in epidemiology, some approaches use a linear function to reconstruct the effective reproduction number (average number of secondary cases per primary case, *R*_*eff*_). Wallinga and Teunis [22] proposed a generic method that requires only case incidence data and the distribution of the serial interval (the time between the onset of symptoms in a primary case and the onset of symptoms of secondary cases) to estimate *R*_*eff*_ over the course of an epidemic. This approach has been improved by numerous authors and applied to real time estimation of *R*_*eff*_ [23–25]. Other authors estimated using mathematical models *R*_*eff*_ for each season [26,27]. To calculate the time-varying infection rate the reconstructed time series of *R*_*eff*_ derived from the notification data can be used [28].

However, the time evolution of *R*_*eff*_ by definition depends not only on the time evolution of the epidemiological parameters but also on the number of susceptibles. More complex approaches have therefore been proposed. These approaches use semi-mechanistic models that incorporate the known compartmental structure of disease transmission but do not specify the form of the transmission rate equation that is estimated based on the data. In an early paper, the force of infection is estimated by neuronal network or kernel regression [29]. Now it is more common to use B-spline [30–33]. An alternative approach is to use diffusion models driven by fractional Brownian motion to model time-varying parameter of major epidemiological significance [34–36]. The models developed assign diffusion processes to the time-varying parameters embedded in a state-space framework. With the Kalman filter, the time-evolution of some key parameters (average transmission rate, mean incubation rate, and basic reproduction rate) were estimated during the course of the HIV/AIDS epidemics in the *Paris region* [34–35]. Dureau et al. [36] generalized this approach using a Bayesian framework with an adjusted adaptive particle Markov chain Monte Carlo algorithm (PMCMC), but only applied to the transmission rate, for short epidemics, with application to the 2009 pandemic flu. Very recently, an algorithm relying on robustly estimating the time-varying infection rate, based on the method of the unknown input observers from control theory, has been proposed [37]. Similarly, an approach for the reconstruction of time-dependent transmission rates, by projecting onto a finite subspace, spanned by Legendre polynomials, has been introduced [38].

In our previous works [34–36], we have introduced an approach for reconstructing the time evolution of some key parameters with just the weak hypothesis according to which they follow a basic stochastic process. The parameter time evolution is estimated solely based on observations of the incidence or the prevalence. Here, we propose to expand this approach to recurrent epidemics over time periods longer than just one season. The underlying idea of this approach is to capture unknown influences by considering time-varying parameters. As with other semi-mechanistic approaches, the key advantage of this approach, for the parameter dynamics, is that it is data-driven, and thus the shape of change does not need to be specified beforehand. We applied our framework both to a toy model, where parameters and observation process are known, and to two real data sets. This allows us to demonstrate that this data-driven approach is very effective for tackling the non-stationarity of recurrent epidemics, even with long time series. It has other benefits too. For instance, with limited access to information, it can capture unknown influences. By so doing, and by analyzing the parameter time evolution, this framework allows a more thorough analysis of the different influences, facilitating their introduction in more complex models with pertinent hypotheses based on observations.

### Models with time-varying parameters

Our approach is based on three main components: an epidemiological model embedded in a state-space framework, a diffusion process for each time-varying parameter and an up-to-date Bayesian inference technique based on adaptive PMCMC.

The main advantage of the state-space framework is the use of two sets of equations, the first set describes the propagation of the disease in the population and the second is for the observation process. This allows for consideration of unknowns and uncertainty both in the epidemiological mechanisms and in the partial observation of the disease:

$$\left\{ \begin{array}{l} \dot{x}(t)=g\left(t,x(t),\theta '(t),u(t)\right)\\ y(t)|x(t)∼f\left(h(x(t)),\theta '(t)\right) \end{array}\right.$$ (1)

The first equation is for the epidemiological model, with *x(t)* representing the state variables (for instance, *S*(*t*) the susceptibles, *I*(*t*) the infectious and *R*(*t*) the removed for the classical SIR model) and *θ'*(*t*) the epidemiological parameters. The second is the observational process defined by probabilistic law *f* and a reporting rate on transformation of some state variable *h*(*x*(*t*)) because we may not be able to directly measure all state variables but just some or a function of them. In these equations, *y*(*t*) are partial observations of *x*(*t*), *u*(*t*) is the process noise describing different form of stochasticity and the observational noise is included in *f*. In our applications, *h*(*x*(*t*)) will be the cumulative sum of new cases over the observation time step, that is generally the quantity observed by Public Health systems.

Considering the time-varying parameters *θ(t)* as a subset of *θ'(t)*, we make the assumption that they evolve more or less randomly and do not follow a defined mathematical function. In the absence of prior information the use of diffusion motion allows us to impose few restrictions on the evolution of *θ*(*t*). We consider that they follow a continuous diffusion process (a discrete diffusion process was used in [35]):

$$\begin{array}{l} d\theta (t)=\sigma dB(t)\\ or\\ d\log(\theta (t))=\sigma dB(t) \end{array}$$ (2)

where *σ* is the volatility of the Brownian process (*dB*(*t*)) and will be estimated during the fitting process. The use of a Brownian process can be viewed as a weak hypothesis for the imposed motion of *θ*(*t*) and the volatility *σ* being a regularized factor. Intuitively, the higher the values of *σ* the larger the changes in *θ*(*t*). The logarithm transformation avoids negative values which have no biological meaning. When prior knowledge on *θ*(*t*) is available this Brownian process can be modified to account for a drift in (2) (see [36]).

For the time-varying parameter, we focus on the parameter of the force of infection classically defined as:

$$\lambda (t)=\beta (t).\frac{S(t).I(t)}{N}$$ (3)

with *β*(*t*) the transmission rate usually defined by a sinusoidal function. The control or the behavior modification can also be taken into account:

$$\lambda (t)=\beta (t).\frac{\left(S{\left(t\right)}^{\varepsilon_{S}(t)}).(I{\left(t\right)}^{\varepsilon_{I}(t)}\right)}{N}$$ (4)

*ε*^i^(*t*) describe the clustering of the population [39,40] but can also describe a reduction in the population due to voluntary avoidance behavior or social distancing. However due to the absence of structural identifiability properties [41, 42] it should be very difficult to estimate simultaneously both *β*(*t*) and *ε*^i^(*t*).

For model estimation we use Bayesian methods, coupling particle filter and MCMC for partially observed stochastic non-linear systems [36,43] (see Methods). The implementation provided in SSM software [36] is used.

## Results

### Reconstructing dynamics and time-varying parameter for a toy SIRS model

**Fig 1:**
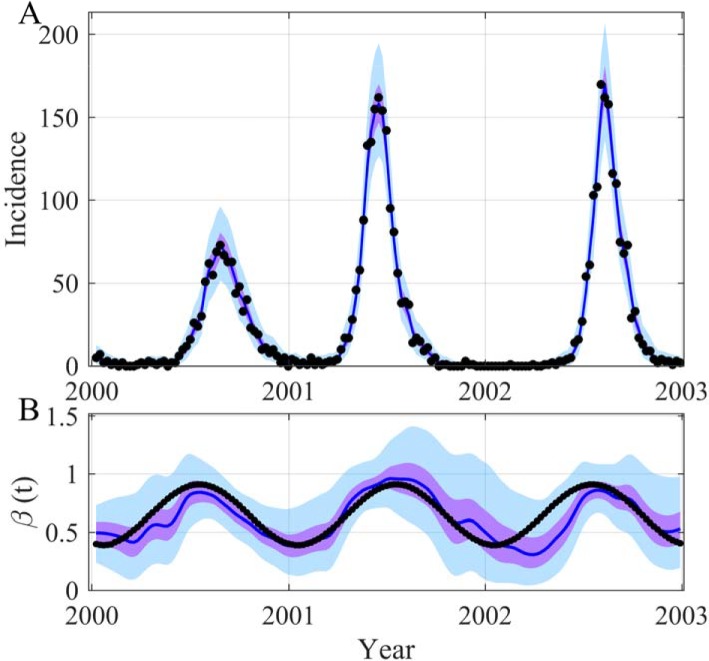
Reconstruction of both the incidence (A) and the time evolution *β*(*t*) (B) for the SIRS model. In (A) the black points are observations generated with a Poisson process with a mean equal to the incidence simulated by the model. In (B) the black points are the true values of *β*(*t*) *= β*_*0*_.(*1 + β*_*1*_ sin(*2π t/365+2πϕ*)). The blue lines are the median of the posterior, the mauve areas are the 50% Credible Intervals (CI) and the light blue areas the 95% CI. For all the figures, the observation process is also applied to the inferred incidence trajectory. The time unit of the model is *day*, the initial date is arbitrary (2000-01-09) and parameters used for the SIRS model are as follows: μ = 1/(50*365), α = 1/(7*365), *γ = 1/14*, *β*_*0*_ *= 0*.*65*, *β*_*1*_ *= 0*.*4*, *ϕ = -0*.*2*, *ρ = 1*, *N = 10000*, *S(0) = 600*, *I(0) = 30*. The prior and posterior distributions of the inferred parameters are in S1 Fig.

**Fig 2:**
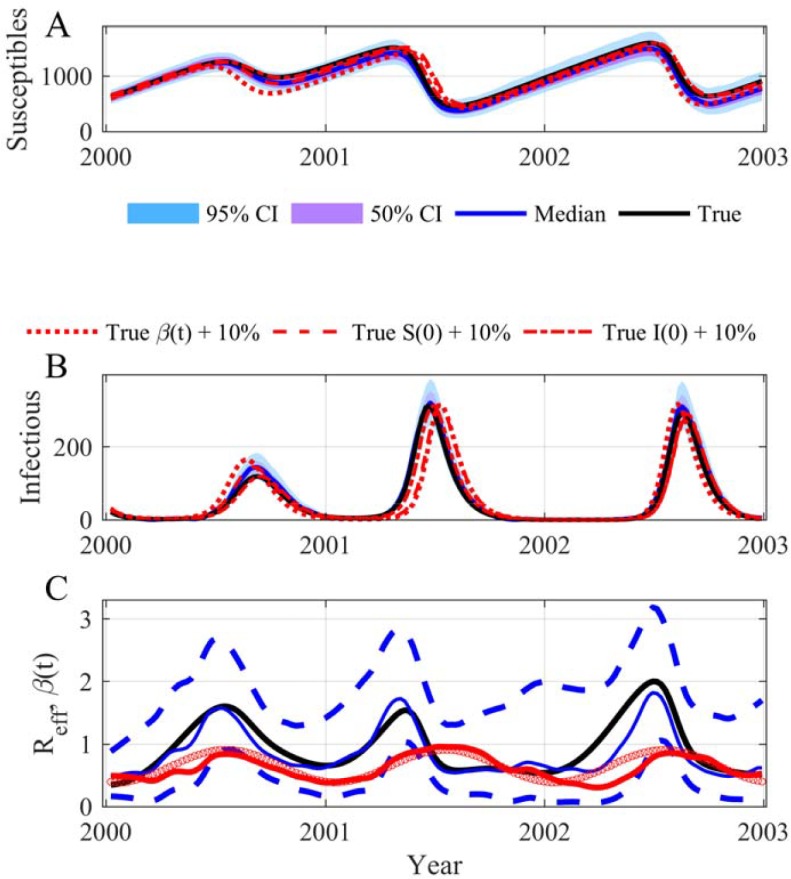
Simulation of the SIRS model: (A) Susceptibles; (B) Infectious; (C) Time evolution of both *R*_*eff*_ and *β(t)*. In (A) and (B) the black lines are the true values, the blue lines are the median of the posterior, the mauve areas are the 50% CI and the light blue areas the 95% CI. In (A) and (B) the susceptibles and infectious trajectories with a modification of 10% of the value of *S*(0), *I*(0) and *β*(*t*) have been added to show the weak sensitivity of the SIRS model to these values. In (C) the black line is the true values of *R*_*eff*_, the blue line is the median of the posterior, and the dashed lines the 95% CI of *R*_*eff*_; the red dot line is the true time evolution of *β*(*t*) and the red line the median of its posterior. Model parameters as in Fig 1: The time unit of the model is *day*, the initial date is arbitrary (2000-01-09) and parameters used for the SIRS model are the following: μ = 1/(50*365), α = 1/(7*365), *γ = 1/14*, *β*_*0*_ *= 0*.*65*, *β*_*1*_ *= 0*.*4*, *ϕ = -0*.*2*, *ρ = 1*, *N = 10000*, *S(0) = 600*, *I(0) = 30*.

**Fig 3:**
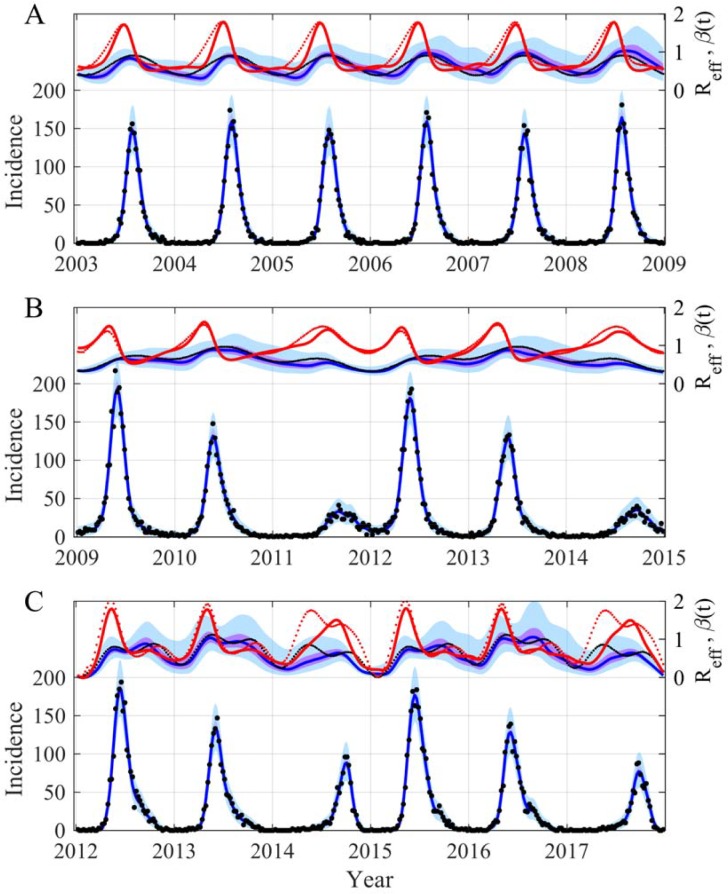
Reconstruction of both the incidence (bottom panel) and the time evolution of *β*(*t*) and *R*_*eff*_ (top panel) for a SIRS model with the initial conditions near the attractor of the dynamics. The true *β* is generated by *β*(*t*) *= β*_*0*_.(*1 + β*_*1*_ sin(*2π t/365+2πϕ) + β*_*2*_ sin(*2πt/*(*3 365*)*+2πϕ)*)*+ β*_*3*_ sin(*2πt/*(*0*.*5 365*)*+2πϕ)*) with in (A) *β*_*1*_ *= 0*.*4*, *β*_*2*_ *= 0*, *β*_*3*_ *= 0*, *S(0) = 911*.*5*, *I(0) = 3*.*5;* in (B) *β*_*1*_ *= 0*.*4*, *β*_*2*_ *= 0*.*3*, *β*_*3*_ *= 0*, *S(0) = 1735*, *I(0) = 20*; and in (C) *β*_*1*_ *= 0*.*1*, *β*_*2*_ *= 0*.*1*, *β*_*3*_ *= 0*.*1*, *S(0) = 3365*, *I(0) = 3*. The other parameters used are as follows: μ = 1/(50*365), α = 1/(7*365), *γ = 1/14*, *β*_*0*_ *= 0*.*65*, *ϕ = -0*.*2*, *ρ = 1*, *N = 10000*. In both panels blue lines are the median of the posterior, the mauve areas are the 50% CI and the light blue areas the 95% CI. In the top panel, the red line is the reconstructed *R*_*eff*_, the points are the true values of *R*_*eff*_ (red) and of *β*(*t*) (black). In the bottom panel the black points are observations generated with a Poisson process. The prior and posterior distributions of the inferred parameters are in S3 Fig.

We start our demonstration by showing that it is possible to reconstruct both the trajectory of a SIRS model (SIRS stands for Susceptibles, Infectious, Removed and Susceptibles again) and that of the sinusoidal transmission rate. In this example, the trajectory of each variable has been simulated with a model for which all the parameters were known. Moreover we also knew the observation process that has generated the data, a Poisson law for the incidence with an observation rate equal to 1. Fig 1 displays the reconstructed trajectories of both the incidence and the transmission rate highlighting the potential of the method. The parameter estimations are in perfect agreement with the values used to generate the observations and the estimation process has correctly converged (S1 and S2 Figs). This clearly demonstrates the feasibility of accurately ascertaining the time evolution of the transmission rate and correctly estimating the *R*_*eff*_ (see Fig 2). It is worth emphasizing that the SIRS model is a complicated example for different reasons. First, even with a constant transmission rate the SIRS model can generate oscillations (damped oscillations, see [17,44]). Secondly, the model trajectories are not very sensitive, a modification of ± 10% can induce minor modifications of the trajectories that are inside or near the 95% CI of our inferences (Fig 2). Moreover in this example we have used initial conditions outside the attractor of the dynamics to generate transients that appear more realistic for real applications, but are more complex to reconstruct. The robustness of our approach has also been tested: (i) using long time series and initial conditions near the attractor (Fig 3A and S3A Fig); (ii) modifying the number of inferring parameters (S4–S6 Figs), for instance estimating just the volatility parameter (S7 and S8 Figs); (iii) considering the possibility of not using the transformation log in the diffusion process (S9 and S10 Figs) and (iv) using a true *β*(*t*) with 2 or 3 periodic components (Fig 3B and 3C and S3B and S3C Fig).

We have also explored the performance of our approach by comparing their inferences to those of the true model. The re-estimation of the true model on its own data is displayed in S11–S13 Figs. Table 1 presents indices of the goodness-of-fit of the true model and models with time-varying *β*(t) with different number of parameters inferred. As expected, the error on *β*(*t*) is smaller when the true equation is used (Table 1). However, regarding the estimated incidence, the true model and our approach give similar results both in terms of mean and variance (Table 1). It could be argued that the price of the flexibility of our approach is a greater variability in some of the trajectory estimations (Table 1). Nevertheless the average dynamics are always estimated correctly.

**Table 1:** Comparison of goodness-of fit indices for different models and different numbers of parameters inferred. The indices are computed on Incidence, *β* and *R*_*eff*_ trajectories: RMSE: root mean square error using the median; MAPE: maximum absolute percentage error using the median; MIQR: mean inter-quartile range. The parameter values used are in the captions of the figures. For comparison purposes we used a stochastic version of the SIRS model with sinusoidal *β* and for all figures the observation process is applied to the inferred incidence trajectory.

**Model**		**Sinusoidal** *β*	**Time-varying** *β***(***t***)**	**Time-varying** *β***(***t***)**	**Time-varying** *β***(***t***)**
**Figure**		**S11 Fig**	**Fig 1**	**S4 Fig**	**S7 Fig**
**# of parameters inferred**		8	7	5	1
Incidence	RMSE	4.18	4.14	4.13	4.12
	MAPE	2.24	2.34	2.39	2.24
	MIQR	1.03	0.95	1.04	1.03
*β(t)*	RMSE	0.07	0.11	0.14	0.13
	MAPE	0.29	0.48	0.52	0.50
	MIQR	0.07	0.43	0.36	0.33
*R*_*eff*_	RMSE	0.06	0.19	0.24	0.24
	MAPE	0.18	0.47	0.50	0.50
	MIQR	0.23	0.64	0.53	0.41

As misspecification is an important problem (*e*.*g*. [45]) we have also compared the performance of our approach to those of a misspecified seasonal SIRS model. We have thus used the example of a sinusoidal *β*(*t*) with two periodic components (see Fig 3B) and computed the indices of the goodness-of-fit of the true model with the SIRS model with 1 year sinusoidal *β*(*t*) and with our time-varying periodic *β*(*t*). The results clearly show that our approach performed better than the misspecified model for the three trajectories analyzed, Incidence, *β and R*_*eff*_ (Table 2). Once again the price of the flexibility of our approach is a greater variability in some of the trajectory estimations. However this is preferable to a large error in the median trajectories as occurred in those observed with the misspecified model (Table 2).

**Table 2:** Comparison of goodness-of fit indices between a misspecified model and a model with a time-varying parameter. The reference is a sinusoidal model with 2 periodic components. The parameter values used are in the captions of Fig 3. The indices are computed on Incidence, *β* and *R*_*eff*_ trajectories: RMSE: root mean square error using the median; MAPE: maximum absolute percentage error using the median; MIQR: mean inter-quartile range. For comparison purposes we used a stochastic version of the SIRS models with sinusoidal *β* and for all figures the observation process is applied to the inferred incidence trajectory.

**Model**		Reference: Sinusoidal *β* with 2 periods (see Fig **3**B caption)	Sinusoidal *β* with 1 year period (see Fig **3**A caption)	**Time-varying** *β* **(***t***) (5)**
**# of parameters inferred**		8 including 4 for *β*	7 including 3 for *β*	5 including 1 (*σ*) for *β*
Incidence	RMSE	4.80	5.21	4.83
	MAPE	2.21	1.98	2.40
	MIQR	0.87	1.03	0.98
*β*(*t*)	RMSE	0.05	0.26	0.08
	MAPE	0.22	1.43	0.27
	MIQR	0.06	0.06	0.28
*R*_*eff*_	RMSE	0.06	0.22	0.08
	MAPE	0.12	0.44	0.20
	MIQR	0.20	0.20	0.46

**Fig 4:**
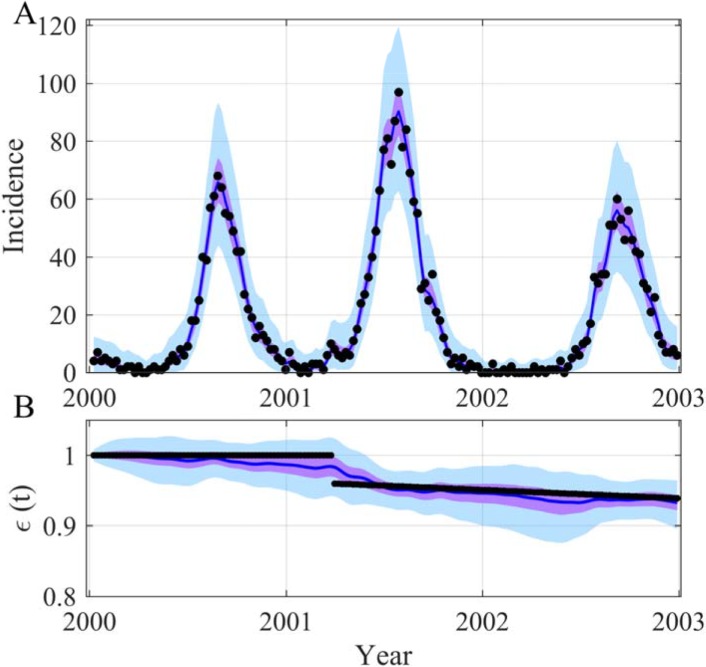
Reconstruction of both the incidence (A) and the time evolution of *ε*_S_(*t*) (B) for the SIRS model. In (A) the black points are observations generated with a Poisson process with a mean equal to the incidence simulated by the model. In (B) the black points are the true value of *ε*_S_(*t*): *ε*_S_(*t*) = *1* and shift to *ε*_S_(*t*) *= 0*.*96 -* (*0*.*012/365*)*(*t—t*_shift_) at *t*_shift_ *= 450* days after *t*_*0*_ in days. The blue lines are the median of the posterior, the mauve areas are the 50% CI and the light blue areas the 95% CI. The time unit of the model is *day*, the initial date is arbitrary (2000-01-09), *β*(*t*) *= β*_*0*_.(*1 + β*_*1*_ sin(*2πt/365+2πϕ)*) and parameters used for the SIRS model are as follows: μ = 1/(50*365), α = 1/(7*365), *γ = 1/14*, *β*_*0*_ *= 0*.*65*, *β*_*1*_ *= 0*.*4*, *ϕ = -0*.*2*, *ρ = 1*, *N = 10000*, *S(0) = 600*, *I(0) = 30*. The prior and posterior distributions of the inferred parameters are in S15 Fig.

Our methodology is also applicable to other more complex or simpler tasks. For instance, it can follow the time evolution of a parameter describing the availability of susceptibles, *ε*_S_(*t*) (Fig 4 and S14 and S15 Figs). Fig 4 shows the accurate reconstruction of the trajectory of the incidence and also of the trajectory of *ε*_S_(*t*) that shifted at a given time point and decreased slightly thereafter. This highlights once again the potential of our approach as it is never easy to estimate a discontinuous dynamic with a continuous process (2).

### Application to real datasets: Flu

**Fig 5:**
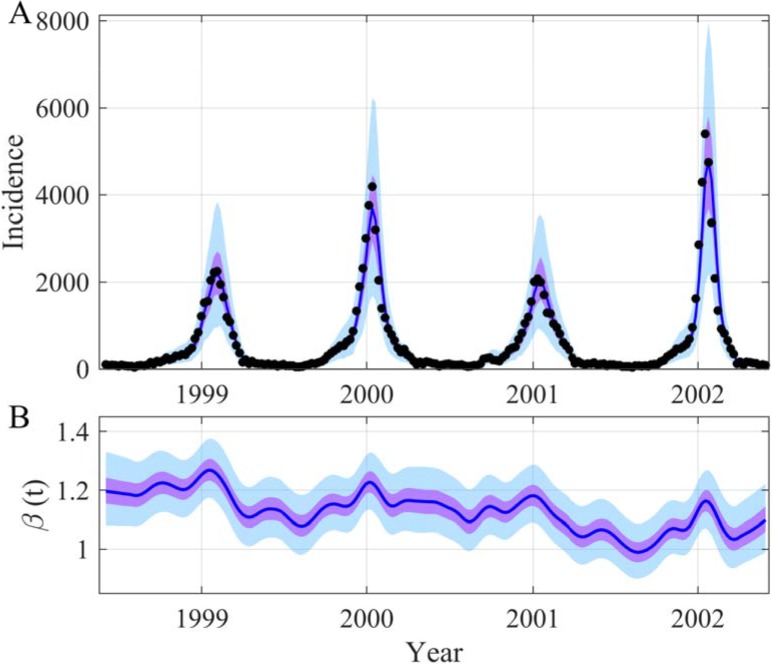
Reconstruction of both the incidence (A) and the time evolution *β*(*t*) (B) in the case of the 1998–2002 seasonal flu epidemics in Israel. In (A) the black points are influenza-like illness incidence collected by Israel’s Maccabi health maintenance organization [46]. The blue lines are the median of the posterior, the mauve areas are the 50% CI and the light blue areas the 95% CI. The prior and posterior distributions of the inferred parameters are in S16 Fig.

In previous works, the dynamics of influenza in Israel have been analyzed using a discrete deterministic SIRS model and weekly data from Israel’s Maccabi health maintenance organization [20,46]. To describe the seasonality of this recurrent epidemic, the authors used a linear model between the transmission rate and local climatic variables, daily temperature and relative humidity [20,46]. We have re-analyzed their dataset (but limited to 1998–2003 due to a modification in the reporting) to reconstruct the time evolution of *β*(*t*). Our results (Fig 5 and S16 Fig) clearly show the potential of our method, highlighting that the *β*(*t*) fluctuations are more irregular and complex than a simple sinusoidal function.

### Application to real datasets: Dengue

**Fig 6:**
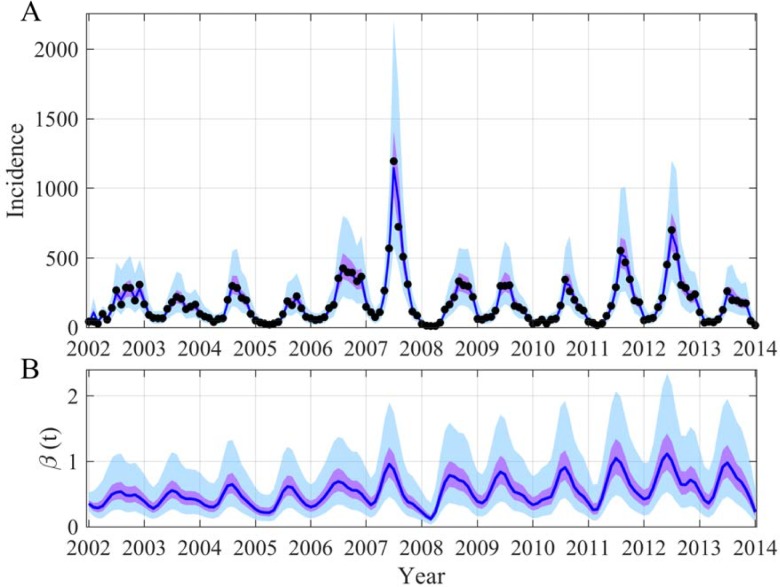
Reconstruction of both the incidence (A) and the time evolution of *β*(*t*) (B) in the case of the 2002–2013 dengue epidemics in the province of Phnom Penh (Cambodia). In (A) the black points are dengue incidence recorded by the Cambodian National surveillance (National Dengue Control Program from the Ministry of Health, see [47]). The blue lines are the median of the posterior, the mauve areas are the 50% CI and the light blue areas the 95% CI. The prior and posterior distributions of the inferred parameters are in S17 Fig.

Our last example is on dengue in Cambodia. Again the idea is to relax the assumption of a sinusoidal *β*(*t*) in a SEIR model. Monthly data from the capital Phnom Penh [47], for which the meteorological data is available from the international airport, was used. We can accurately describe the 12 year time series and reconstruct the time evolution of *β*(*t*) (Fig 6 and S17 Fig). Our results stress that the *β*(*t*) oscillations are more complex than a simple sinusoidal function. Sometimes bi-modality occurs over one season. In general one observes a fast growth of *β*(*t*) and a slow decrease. Moreover the amplitude of the *β*(*t*) varies from year to year, perhaps depending on the fluctuations in the mosquito population and in the environment. Interestingly the peak in *β*(*t*) appears 1 to 2 months before the incidence peak. This delay can be explained by the extrinsic incubation period and might be used in a warning system.

**Fig 7:**
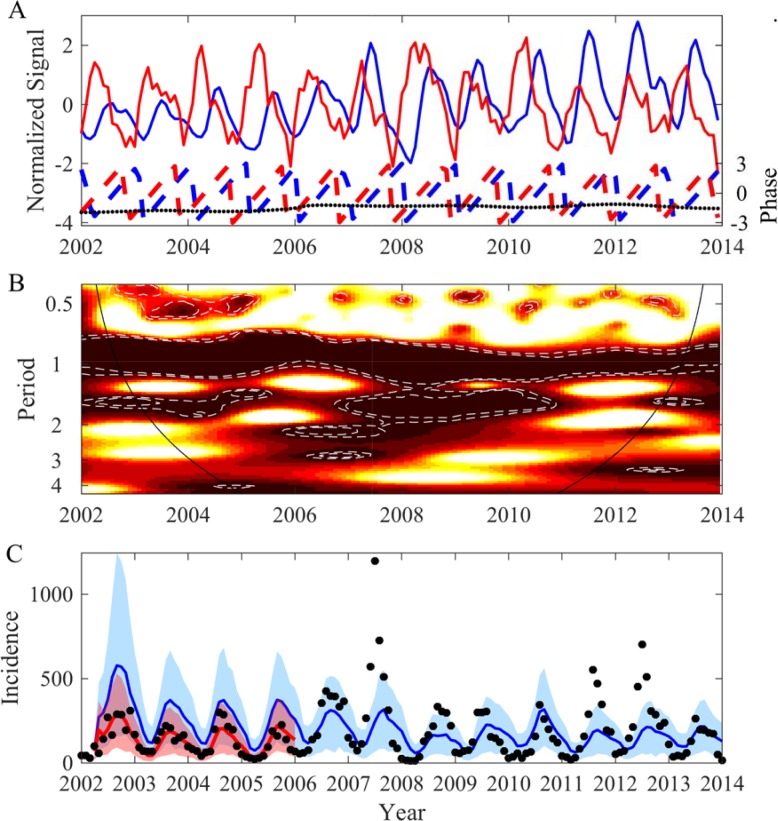
Association between dengue transmission rate and monthly average maximum temperature in the province of Phnom Penh (Cambodia). (A) Time evolution of the normalized median of *β*(*t*) (blue line) and average temperature (red line) as well as the evolution of their phase computed based on wavelet decomposition (see Method and [48,70]), blue dashed line for the normalized *β*(*t*), red dashed line for the normalized averaged temperature and black dotted line for their phase difference. (B) Wavelet coherence (see Method and [48,70]) between the reconstructed *β*(*t*) and average temperature. The colors code for low values in white to high values in dark red. The white dashed lines show the 90% and the 95% CI computed with adapted bootstrappes [71]. (C) Model simulations using a linear model describing *β*(*t*) with monthly average maximum temperature (S18 Fig) and monthly average minimum temperature (S19 Fig) (β(*t*) *= a*_*0*_ *+ a*_*1*_.*MaxTemp*(*t*)+*a*_*2*_.*MinTemp*(*t*)). The red line is the median of the posterior and the red area is the 95% CI when parameters are estimated for the period 2002–2005. The blue line is the median of the posterior and the light blue area is the 95% CI when parameters are estimated for the full time period. The black points are dengue incidence recorded by the Cambodian National surveillance [47].

To explain the *β*(*t*) oscillations we have explored the potential effects of local and global climatic variables using wavelet decomposition [48] as one of our main underlying hypotheses is non-stationarity. We observed very significant coherency between *β*(*t*) and climate for the local climate for the seasonal mode (Fig 7 and S18–S20 Figs) and also for the 2–3 year components with global climatic variable (S21 Fig). Thus, the rhythm of *β*(*t*) can be explained perfectly by climatic factors. Nevertheless, again mainly due to large non-stationarity, by using solely one or two climatic variables we are able to correctly describe dengue evolution in the short-term (Fig 7C, red area) but not over a large time period (Fig 7C, blue area). This reflects the complexity of such a disease where the ecology of the vectors, the environment, the climate, the immune status of the human population and its behavior are all involved. This large non-stationarity association between dengue and climatic factors has recently been demonstrated using statistical models (dynamic generalized linear models) and data from a medium-sized city in Colombia [49]. The authors showed that dengue cases correlate with climatic variables (temperature, rainfall, solar radiation and relative humidity) but these correlations change over time, some intervals showing a positive association, while in others the association is negative [49]. The non-stationarity association between dengue and climate may be explained by the fact that a climatic variable has different effects depending on the biological cycle of the pathogen or of the vector. Moreover the effects of one climatic variable can also depend on other climatic variables potentially enhancing the non-stationarity association.

## Discussion

As there remain numerous uncertainties during the course of each epidemic, we are increasingly aware of the importance of developing adequate statistical and mathematical tools. Such tools need to take account of the time-varying nature of the underlying ecological and biological mechanisms as well as social and behavioral influences involved in an epidemic. Because of this, time-varying parameters modeled with a diffusion process, that track epidemiological patterns and update the key parameters according to data appear to be a worthwhile approach. Indeed developing a more complex model would be difficult considering the relative paucity of available data.

We propose a flexible modeling framework that encompasses time-varying aspects of the epidemic. It does this via diffusion process equations for time-varying parameters and also considers uncertainty associated with key parameters and data. This data-driven framework for time-varying parameters has been coupled with simple stochastic models and a robust Bayesian procedure for inference. To test its efficiency, our proposed methodology was first applied to a toy model and then to real epidemiological examples.

Our results clearly demonstrate the potential of our framework. Firstly, our methodology was able to accurately reconstruct both the incidence and the sinusoidal transmission rate of a simple SIRS model just based on noisy observations (Figs 1–4 and S4,S5,S7,S9 and S14 Figs). Based on these reconstructions one can also closely estimate *R*_*eff*_ which is one of the key relevant epidemiological parameters. Our results also highlight the flexibility of our developed methodology. It can reconstruct the time evolution of a shifting parameter (*ε*_S_(*t*), see Fig 4 and S14 Fig) as well as an oscillating parameter that influences the nonlinear part of the model (*β*(*t*), see Figs 1–3 and S4,S5,S7 and S9 Figs). The comparison using goodness-of-fit indices with the inferred true model allows us to highlight the fact that our methodology performs as well for the observed incidence. Its flexibility results in greater variability in some other trajectories mainly *β*(*t*) and *R*_*eff*_ (Table 1). Moreover, in the absence of knowledge of the true evolution of the transmission rate, our approach appears to capture the dynamic observed more accurately than a misspecified model (Table 2). Secondly, applied to real datasets, our framework is able, based solely on simple stochastic models, to reconstruct complex epidemics such as flu or dengue over long time periods (Figs 5 and 6). In such cases, the reconstruction of the time evolution of the transmission rate clearly stresses that, on real datasets, it is difficult to assimilate the dynamic of this parameter as a simple sinusoidal function. It is more irregular in amplitude and sometimes multi-modal over one season.

Considering the paucity of information available regarding the complexity of the mechanisms involved during an epidemic, describing and fitting a full model for a given transmissible disease is always challenging. Our data-driven methodology can be used as a first step towards a better understanding of a complex epidemic, where data is limited or lacks certainty. Indeed most of the unknowns and uncertainties can be put into time-varying parameters. The potential effects of all these uncertainties can then be explored by analyzing the reconstructed time evolution of the time-varying parameters. See Fig 7 for such preliminary analysis of dengue in Phnom Penh. This allows a more thorough analysis of the influences and the interactions between both the human behavior and complex environmental drivers. In a recent paper [50], the authors reviewed evidence of interactions between seasonal influenza virus and other pathogens (bacteria or virus). They concluded that it is important to incorporate these different coinfecting pathogens in models of seasonal flu in order to get a better estimate of the burden of influenza. Our framework could be an alternative to the development of complex models with all the potential interactions between pathogens and to estimate the strength of the interactions. After reconstructing the time evolution of the transmission rate the statistical association between the coinfecting pathogens and the transmission rate could be tested. This screening may facilitate the construction of more complex models that could incorporate only the most significant coinfecting pathogens in the seasonal flu model.

Our methodology also has other advantages. Taking account of the simplicity of the model used, and the fact that weak hypotheses on the dynamics of the time-varying parameters have been included, our proposed methodology can retrospectively test the impact of interventions. This has previously been done in the case of HIV epidemics [34–35], where it was hypothesized that the reduction in the transmissibility was due to a modification of the sexual behavior in the population and the increase in the seropositive period duration due to the introduction of the first antiviral treatments. Evaluation of interventions has also been done recently in the case of the Ebola epidemic in West Africa [51]. The relative simplicity of our methodology is also suitable for short-term predictions and it can then easily be used to predict an epidemic in real time. Starting with a given estimated state defining the system, the fitting process can be run again each time new data is available and the new posteriors are used for new predictions [36]. This can inform public health decisions and indeed has been done recently to great effect in the case of the Ebola epidemics in West Africa [52].

A major challenge in model fitting is the reliability of data collected and also the non-identifiability of the mechanistic models that always have very rich dynamical behavior. The question of identifiability is too often avoided in epidemiological models applied to a topical Public Health issues. There is, however, considerable literature on this subject (*e*.*g*. [41,42,53–55]). Identifiability is not evident even for a simple seasonal SIR model [56]. To solve this problem one can fit a combination of parameters or fix some of them (the population size for instance) [57]. In our applications there is a clear limitation due to practical non-identifiability of reporting rate and initial conditions. To fix these problems we have used informative priors (see Method). Using informative priors or fixing some parameters gives very similar results (compare Fig 1 and S4–S7 Figs). Related to this is the misspecification of models [45]. In our cases, as with other semi-mechanistic models the time-varying parameter methodology captures some of the information in the data but not in the mechanistic part of the model. If the model is misspecified due to lack of precision, it compensates for it and the dynamics of *β*(*t*) will drive improvements in the model to make it more complex and realistic (Table 2). If the model is misspecified to the extent that it creates mechanisms that do not exist, the reconstructed *β*(*t*) would compensate for these effects but it will be harder to interpret.

In this work we have used simple mechanistic models. The proposed methodology is not limited to simple models. For instance, a two-strain dengue model has also been tested. In this case the main problem was linked to the unavailability of both seroprevalence and incidence for each strain. Indeed, one of the major difficulties with these multi-strain models is the identification of the initial conditions (*e*.*g*. [58]). Nevertheless it is worth emphasizing that the Bayesian inference method used in our framework, PMCMC, the approximation of the likelihood is limited for a large number of parameters and/or equations [59]. In such cases testing other methodologies like ABC [60,61] is advisable.

It is always difficult to fit complex models with rich behaviors based on very limited information. In this regard we agree with Metcalf et al. [62] who stressed that nowadays we need seroprevalence studies to quantify the immunological status of the population, because in most cases the magnitude of the outbreak is difficult to evaluate without precise seroprevalence data.

To tackle the uncertainty and the non-stationarity of epidemics, our methodology, although it appears non-standard, makes important progress towards a better understanding of the mechanisms responsible for disease propagation. We believe that, should it form part of the development of the next predictive tools for Public Health, it will make a significant contribution to improving the understanding and control of infectious diseases in our increasingly uncertain world.

## Methods

### Models

#### SIRS model

Our first model is a classical SIRS model with an observation rate ρ *= 1* and Poisson law as the observational process:

$$\begin{array}{l} \dot{S}=\mu .\left(N-S\right)-\beta (t).S.I/N+\alpha .R\\ \dot{I}=\beta (t).S.I/N-\left(\gamma +\mu \right).I\\ \dot{C}=\beta (t).S.I/N\\ \dot{R}=\gamma .I-\left(\alpha +\mu \right).R \end{array}$$ (5)

where *S*, *I* and *R* are the susceptibles, the infectious and the removed respectively, the transmission rate *β(t) = β*_*0*_.*(1 + β*_*1*_ sin*(2πt/365+2πϕ)*, 1/*α* is the average duration of immunity, *γ* is the recovery rate and *μ* is the recruitment or mortality rate. In (5), *C* is the number of new cases, then *h*(*x*(*t*)) is the cumulative sum of *C*(*t*) over the observation time step, 7 days. With this model *R*_*eff*_(*t)* = *β*(*t)*.*S*(*t)/*(*N*.*γ)*. The parameter values are in the caption of Fig 1. For the fit of our simulated data, Gaussian priors are used for epidemiological parameters (α and γ). Initially non-informative priors were used for the volatility *σ*, the reporting rate ρ and the initial conditions *γ*(0) by *β*(0) but to reduce problems linked to practical non-identifiability materialized by correlation between some estimates, informative priors were used for ρ (see S1 Fig). Some other simulations have been done fixing *β*(0) and ρ or just inferring *σ* (see S4–S8 Figs).

#### SIRS flu model

For analyzing Israel flu data we have used a continuous SIRS model identical to (5), we simply added imported infectious people *i* in the force of infection:

$$\lambda (t)=\beta (t).\frac{S(t).(I(t)+i)}{N}$$ (6)

The initial guess values for the parameters are from [46]. In this example the observational process is a Negative-Binomial law with an over-dispersion parameter equal to 0.05 and the reporting rate ρ = 0.15 [46]. For the fit, Gaussian priors are used for epidemiological parameters (*i*, α, γ) and non-informative priors for the volatility *σ* and the initial conditions (*S*(0), *I*(0)). S16 Fig displays the priors and the posteriors.

#### SEIR one strain dengue model

To describe the dengue epidemics, taking account of the available data for Phnom Penh, we have fitted a one strain model using a SEIR model:

$$\begin{array}{l} \dot{S}=\mu .\left(N-S\right)-\beta (t).S.(I+i)/N\\ \dot{E}=\beta (t).S.(I+i)/N-\left(\delta +\mu \right).E\\ \dot{I}=\delta .E-\left(\gamma +\mu \right).I\\ \dot{R}=\gamma .I-\mu .R \end{array}$$ (7)

where *E* is for infected but not yet infectious, *β*(*t*) is the transmission rate, 1/*δ* is the average duration of the latent period, *γ* is the recovery rate and *μ* is the recruitment or mortality rate. The initial guess for parameter values comes from the literature [63]. In this example the observational process is a Negative-Binomial law with an over-dispersion parameter equal to 0.05 and the reporting rate ρ has been estimated using a narrow Gaussian prior. Non-informative priors are used for the volatility *σ*, initial condition for infected *E*(0) and imported infectious people *i*. Gaussian priors are used for other parameters and initial conditions. When *E*(0) is fitted, *I*(0) is estimated as a steady-state value *I*(0) *= δ*.*E*(0)/(*γ+μ*). S17 Fig. displays the priors and the posteriors.

### Inference

#### Stochastic framework

Due to the use of a diffusion Eq (2) for the dynamic of the time-varying parameters, the stochastic versions of the previous models have been fitted. Thus the models are considered in a stochastic framework in which the compartments are discrete and the number of reactions occurring in a time interval *dt* is approximated by a multinomial distribution. It is fully described in [64,65].

#### SMC algorithm as implemented in SSM [65]

In a model with *n* observations and *J* particles. *L* is the model likelihood *p*(*y*_1:*n*_|*θ*). $W_{k}^{\left(j\right)}$ is the weight and $x_{k}^{\left(j\right)}$ is the state associated to particle *j* at iteration *k*.

1. Set $L=1,\;W_{0}^{\left(j\right)}=1/J$.

2. Sample ${\left(x_{0}^{\left(j\right)}\right)}_{j=1:J}$ from *p*(*x*_0_|*θ*)

3. **for** (*k* = 0:*n*−1) **do**

4.     **for** (*j* = 1:*J*) **do**

5.         Sample $x_{k+1}^{\left(j\right)}$ from $p(x_{k+1}|x_{k}^{\left(j\right)},\theta )$

6.         Set $\alpha^{\left(j\right)}=p(y_{k+1}|x_{k+1}^{\left(j\right)},\theta )$

7.     **end for**

8.     Set Wk+1(j)=α(j)∑l=1Jα(l) and $L=L\frac{1}{J}\sum_{l=1}^{J}\alpha^{\left(l\right)}$

9.     Resample ${\left(x_{0:k+1}^{\left(j\right)}\right)}_{j=1:J}$ from $W_{k+1}^{\left(j\right)}$.

10. **end for**

#### Estimation with particle Markov Chain Monte Carlo (PMCMC)

Since the epidemiological propagation models are considered in a stochastic framework, their likelihood is intractable and it is estimated with particle filtering methods (Sequential Monte Carlo, SMC). With a given set of parameters, the SMC algorithm reconstructs sequentially the trajectory of the state variables and the time-varying parameters, and computes the associated likelihood. Firstly, the distribution of the initial conditions of the system is approximated with a sample of particles. Then, at each iteration, the particles are projected according to the propagation model up to the next observation point, they receive a weight reflecting the quality of their prediction compared to the observation and the total likelihood is updated. A resampling step using the weights is performed before the next iteration, in order to discard the trajectories associated with low weight particles.

In order to estimate the parameters of the system, the particle filter is embedded in a Markov Chain Monte Carlo framework, leading to the PMCMC algorithm [43]. More precisely, the likelihood estimated by SMC is used in a Metropolis Hasting scheme (particle marginal Metropolis Hastings) [43]. The proposal distribution is a Gaussian whose co-variance matrix is adapted following the framework described in [65].

The starting point of the MCMC chain is initialized using optimal values obtained from the KSimplex algorithm on a large number of parameter sets. Then, a pre-adaptation of the proposal co-variance matrix is performed with Kalman MCMC (KMCMC). Each time the idea relies on less computationally intense algorithms in order to facilitate the exploration of parameter space. But as we use stochastic models we approximate the likelihood using the extended Kalman filter both in the simplex algorithm (KSimplex) [65] and in the MCMC (KMCMC) [36]. Then the adaptive PMCMC is executed on the output of the KMCMC with 100000 iterations and 10000 particles for the final figures. For instance, the results such as those of Fig 1 take less than 24 hours, on a blade server from PowerEdge M-Series with 40 processor cores.

#### PMCMC algorithm as implemented in SSM [65]

In a model with *n* observations and *J* particles.

*q*(.|*θ*^(*i*)^) is the transition kernel

1. Initialize *θ*^(0)^

2. Using SMC algorithm, compute $\hat{p}(y_{1:n}|\theta^{\left(0\right)})$ and sample $x_{0:n}^{\left(0\right)}$ from $\hat{p}(x_{0:n}|{y_{1:n},\theta }^{\left(0\right)})$

3. **for** (*i* = 0:*N*−1) **do**

4.     Sample θ* from *q*(.|*θ*^(*i*)^)

5.     Using SMC algorithm, compute $L(\theta^{*})=\hat{p}(y_{1:n}|\theta^{*})$ and sample $x_{0:n}^{*}$ from $\hat{p}(x_{0:n}|{y_{1:n},\theta }^{*})$

6.     Accept θ* (and $x_{0:n}^{*}$) with probability $1\wedge \frac{L(\theta^{*})p(\theta^{*})q(\theta^{\left(i\right)}|\theta^{*})}{L(\theta^{\left(i\right)})p(\theta^{\left(i\right)})q(\theta^{*}|\theta^{\left(i\right)})}$

7.     If accepted, *θ*^(*i*+1)^= θ* and $x_{0:n}^{\left(i+1\right)}=x_{0:n}^{*}$. Otherwise, *θ*^(*i*+1)^= *θ*^(*i*)^ and $x_{0:n}^{\left(i+1\right)}=x_{0:n}^{\left(i\right)}$.

8. **end for**

In order to assess convergence of the chain, the visual inspection of the chains (*e.g.* S2 Fig or S8 Fig) was complemented by diagnosis provided in the Coda package in R [66]. Due to the large computational cost of the algorithm, we did not run multiple independent chains, rather we relied on diagnosis using one MCMC chain and testing its stationarity: Geweke diagnosis [67] and Heidelberger and Welch’s diagnosis [68]. The results are presented in S1 and S2 Tables.

### Wavelet analysis

Among the various approaches developed to study nonstationary data, wavelet analysis is probably the most efficient. In particular, this method gives us the possibility of investigating and quantifying the evolution in time of the periodic components of a time series (see [69]). Wavelets constitute a family of functions derived from a single function, the ‘‘mother wavelet”, *Ψ*_*a*,*τ*_*(t)*, that can be expressed as the function of two parameters, one for the time position *τ*, and the other for the scale of the wavelets *a*, related to the frequency. More explicitly, wavelets are defined as:

$$\Psi_{a,\tau }(t)=\frac{1}{\sqrt{a}}\psi \left(\frac{t-\tau }{a}\right)$$

The wavelet transform of a time series *x*(*t*) with respect to a chosen mother wavelet is performed as follows:

$$W_{x}(a,\tau )=\frac{1}{\sqrt{a}}.\int_{-\infty }^{+\infty }x(t).\Psi_{}^{*}\left(\frac{t-\tau }{a}\right).dt=\int_{-\infty }^{+\infty }x(t).\Psi_{a,\tau }^{*}.dt$$

where * denotes the complex conjugate form. The wavelet transform *W*_*x*_(*a*,*τ*) represents the contribution of the scale *a* to the signal at different time positions *τ*. The computation of the wavelet transform is done along the signal *x*(*t*) simply by increasing the parameter *τ* over a range of scales *a* until all coherent structures within the signal can be identified. Here, as mother wavelet, we have used the Morlet wavelet [69].

With the wavelet approach, we can estimate the repartition of variance at different scale *a* and different time location *τ*. This is known as the wavelet power spectrum: *S*_*x*_(*a*,*τ*) *= | W*_*x*_(*a*,*τ) |*^*2*^. An important point with the continuous wavelet is that the relationship between the wavelet frequency *f*_*0*_ and the wavelet scale *a* can be derived analytically. For the Morlet wavelet this relationship is given by:

$$\frac{1}{f}=\frac{4\pi a}{f_{0}+\sqrt{2+f_{0}^{2}}}$$

Then when *f*_*0*_ *= 2π*, the wavelet scale *a* is inversely related to the frequency, *f ≈ 1/a*. This greatly simplifies the interpretation of the wavelet analysis and one can replace, on all equations, the scale *a* by the frequency *f* or the period *1/f*.

To determine the statistical relationship between two time series, wavelet coherence can be computed (*e*.*g*. [48,70]):

$$R_{x,y}(f,\tau )={\left(\frac{{\left|\langle W_{x,y}(f,\tau )\rangle \right|}^{2}}{{\left|\langle W_{x}(f,\tau )\rangle \right|}^{2}.{\left|\langle W_{y}(f,\tau )\rangle \right|}^{2}}\right)}^{1/2}$$

where the angle brackets around terms indicate smoothing in both time and frequency, *W*_*x*_(*f*,*τ*) is the wavelet transform of series *x*(*t*), *W*_*y*_(*f*,*τ*) is the wavelet transform of series *y*(*t*), and *W*_*x*,*y*_(*f*,*τ*) is the cross-wavelet spectrum. The values of wavelet coherence are between *0 < R*_*x*,*y*_(*f*,*τ*) *< 1*. The wavelet coherency is equal to 1 when there is a perfect linear relation at particular time and scale between the two signals, and equal to 0 if *x*(*t*) and *y*(*t*) are independent.

To complement this, phase analysis can be used to characterise the association between signals (*e*.*g*. [48,70]). The phase difference provides information on the sign of the relationship (*i*.*e*., in phase or out of phase) and can be computed, for complex mother wavelet, with the wavelet transform *W*_*x*_(*f*,*τ*) as:

$$\phi_{x}(f,\tau )=tan^{-1}\frac{Im(W_{x}(f,\tau ))}{Re(W_{x}(f,\tau ))}$$

Similarly with the cross-wavelet transform *W*_*x*,*y*_(*f*,*τ*) the phase difference between the two time series can be computed:

$$\phi_{x,y}(f,\tau )=tan^{-1}\frac{Im(W_{x,y}(f,\tau ))}{Re(W_{x,y}(f,\tau ))}$$

## Supporting information

S1 Code Examples of model files and code for using SSM. (ZIP)

S1 Table Test of the MCMC chains: Geweke diagnosis [67] that tests for the non-stationarity of the chains, the parameter means computed using the first 10% and the last 50% of the chain are compared through a Z-score (stationarity is not rejected if the Z-scores are below the critical values at 5%, NS (non-significant) in the Table). The test was implemented with the Coda package in R [66].
(PDF)

S2 Table Test of the MCMC chains: Heidelberger and Welch’s diagnosis [68] that tests for the non-stationarity of the chains (NS (non-significant) in the Table meaning stationarity is not rejected at the 5% level). The test was implemented with the Coda package in R [66].
(PDF)

S1 Fig Prior and posterior distributions for the SIRS model inferences of Fig 1. *I*(0), *S*(0) initial values, *β*(0) initial value of *β*(*t*), 1/*α* is the average duration of immunity, *γ* is the recovery rate, ρ is the reporting rate and *σ* is the volatility of the Brownian process of *β*(*t*). The blue distributions are the priors and the discrete histograms are the posteriors. The medians of the prior distributions for *I*(0), *S*(0), *β*(0), 1/*α*, 1/*γ* and ρ are the “true values” used for the simulations of the observed incidences.
(PDF)

S2 Fig The traces of the MCMC chain for the SIRS model inferences of Fig 1. *I*(0), *S*(0) initial values, *β*(0) initial value of *β*(*t*), 1/*α* is the average duration of immunity, *γ* is the recovery rate, ρ is the reporting rate and *σ* is the volatility of the Brownian process of *β*(*t*).
(PDF)

S3 Fig Prior and posterior distributions for the SIRS model inferences displayed on Fig 3 when the initial conditions are near the attractor of the dynamics. A/ Observed data generated with a SIRS model and a sinusoidal *β* with 1 periodic component (5). B/ Observed data generated with a SIRS model and a sinusoidal *β* with 2 periodic components. In A/ and B/, *I*(0), *S*(0) are initial values, 1/*α* is the average duration of immunity, *γ* is the recovery rate and *σ* is the volatility of the Brownian process of *β*(*t*). C/ Observed data generated with a SIRS model and a sinusoidal *β* with 3 periodic components, *σ* the volatility of the Brownian process of *β*(*t*) is the only parameter inferred. The blue distributions are the priors and the discrete histograms are the posteriors. The medians of the prior distributions are the “true values” used for the simulations of the observed incidences.
(PDF)

S4 Fig Reconstruction of both the incidence (A) and the time evolution *β*(*t*) (B) for the SIRS model as in Fig 1 but only 5 parameters have been inferred, *β*(0) and ρ were fixed. Model parameters as in Fig 1 and S6 Fig.
(PDF)

S5 Fig Simulation of the SIRS model when the initial conditions are near the attractor of the dynamics and just 5 parameters inferred: (A) Susceptibles; (B) Infectious; (C) Time evolution of both *R*_*eff*_ and *β*(*t*). In (A) and (B) the black lines are the true values, the blue lines are the median of the posterior, the mauve areas are the 50% CI and the light blue areas the 95% CI. In (C) the black line is the true values of *R*_*eff*_, the blue line is the median of the posterior, and the dashed lines the 95% CI of *R*_*eff*_; the red dot line is the true time evolution of *β*(*t*) and the red line the median of its posterior. Model parameters as in Fig 1 and S6 Fig.
(PDF)

S6 Fig Prior and posterior distributions for the SIRS model inferences of S4 Fig. *I*(0), *S*(0) initial values, 1/*α* is the average duration of immunity, *γ* is the recovery rate and *σ* is the volatility of the Brownian process of *β*(*t*). The blue distributions are the priors and the discrete histograms are the posteriors. The medians of the prior distributions for *I*(0), *S*(0), 1/*α* and 1/*γ*, are the “true values” used for the simulations of the observed incidences.
(PDF)

S7 Fig Reconstruction of both the incidence (A) and the time evolution *β*(*t*) (B) for the SIRS model as in Fig 1 but *σ* the volatility of the Brownian process of *β*(*t*) is the only parameter inferred. Model parameters as in Fig 1 and S8 Fig.
(PDF)

S8 Fig The trace of the MCMC chain and the prior and posterior distributions for the SIRS model inferences of S7 Fig when *σ* is the volatility of the Brownian process of *β*(*t*) is the only parameter inferred. (PDF)

S9 Fig Reconstruction of both the incidence (A) and the time evolution *β*(*t*) (B) for the SIRS model as in S4 Fig but the logarithm transformation of the Brownian process of *β*(*t*) is not used: *dθ*(*t*) = *σ*.*dB*(*t*). Model parameters as in Fig 1 and S4 Fig.
(PDF)

S10 Fig As in S6 Fig but the logarithm transformation of the Brownian process of *β*(*t*) is not used: *dθ*(*t*) = *σ*.*dB*(*t*). (PDF)

S11 Fig Reconstruction of both the incidence (A) and the time evolution *β*(*t*) (B) with the true SIRS model. In (A) the black points are observations generated with a Poisson process with a mean equal to the incidence simulated by the model. In (B) the black points are the true values of *β*(*t*) *= β*_*0*_.(*1 + β*_*1*_ sin(*2πt/365+2πϕ*)). The blue lines are the median of the posterior, the mauve areas are the 50% Credible Intervals (CI) and the light blue areas the 95% CI. For all the figures, the observation process is also applied to the inferred incidence trajectory. The time unit of the model is *day*, the initial date is arbitrary (2000-01-09) and parameters used for the SIRS model are as follows: μ = 1/(50*365), α = 1/(7*365), *γ = 1/14*, *β*_*0*_ *= 0*.*65*, *β*_*1*_ *= 0*.*4*, *ϕ = -0*.*2*, *ρ = 1*, *N = 10000*, *S(0) = 600*, *I(0) = 30*. The prior and posterior distributions of the inferred parameters are in S13 Fig
(PDF)

S12 Fig Simulation of the true SIRS model: (A) Susceptibles; (B) Infectious; (C) Time evolution of both *R*_*eff*_ and *β*(*t*). In (A) and (B) the black lines are the true values, the blue lines are the median of the posterior, the mauve areas are the 50% CI and the light blue areas the 95% CI. In (C) the black line is the true values of *R*_*eff*_, the blue line is the median of the posterior, and the dashed lines the 95% CI of *R*_*eff*_; the red dot line is the true time evolution of *β*(*t*) and the red line the median of its posterior. Model parameters as in S11 Fig and S13 Fig.
(PDF)

S13 Fig Prior and posterior distributions for the true SIRS model inferences of S11 Fig. *I*(0), *S*(0) initial values, *β*_*0*_, *β*_*1*_, *ϕ*, the parameters of the sinusoidal *β*, 1/*α* is the average duration of immunity, *γ* is the recovery rate and ρ is the reporting rate. The blue distributions are the priors and the discrete histograms are the posteriors. The medians of the prior distributions are the “true values” used for the simulations of the observed incidences.
(PDF)

S14 Fig Simulation of the SIRS model: (A) Susceptibles; (B) Infectious; (C) Time evolution of both *R*_*eff*_ and *ε*_S_(*t*). In (A) and (B) the black lines are the true values, the blue lines are the median of the posterior, the mauve areas are the 50% CI and the light blue areas the 95% CI. In (C) the black line is the true values of *R*_*eff*_, the blue line is the median of the posterior and the dashed lines the 95% CI of *R*_*eff*_; the red dot line is the true time evolution of *ε*_S_(*t*) and the red line the median of its posterior. Model parameters as in Fig 4 and S15 Fig.
(PDF)

S15 Fig Prior and posterior distributions for the SIRS model inferences of Fig 4. *I*(0), *S*(0) initial values, 1/*α* is the average duration of immunity, *γ* is the recovery rate and *σ* is the volatility of the Brownian process of *ε*_S_(*t*). The blue distributions are the priors and the discrete histograms are the posteriors. The medians of the prior distributions for *I*(0), *S*(0), 1/*α* and 1/*γ*, are the “true values” used for the simulations of the observed incidences.
(PDF)

S16 Fig Prior and posterior distributions for the parameters of the SIRS flu model. *I*(0), *S*(0) initial values expressed in percentage of the population *N*, *β*(0) initial value of *β*(*t*), *i* imported infectious, 1/*α* is the average duration of immunity, *γ* is the recovery rate and *σ* is the volatility of the Brownian process of *β*(*t*). The blue distributions are the priors and the discrete histograms are the posteriors. Prior values are adapted from [46].
(PDF)

S17 Fig Prior and posterior distributions for the parameters of the SEIR dengue model. *E*(0), *S*(0) expressed in percentage of the population *N*, *β*(0) the initial value of *β*(*t*), *γ* is the recovery rate, *i* imported infectious, 1/*α* is the average duration of immunity, *σ* is the volatility of the Brownian process of *β*(*t*) and ρ the reporting rate. The blue distributions are the priors and the discrete histograms are the posteriors. Prior values are adapted from [63].
(PDF)

S18 Fig Association between dengue transmission rate and monthly average maximum temperature recorded at the Phnom Penh International Airport (Cambodia). (A) Time evolution of the normalized *β*(*t*) (blue line) and normalized average temperature (red line). (B) and (C) Wavelet Power Spectrum (WPS) [48,70] of the two time series. The graph on the right shows the average WPS. (D) Wavelet coherence [48,70] between the reconstructed *β*(*t*) and average temperature. In (B), (C) and (D) the colors code for low values in white to high values in dark red. The dashed lines show the 95% CI computed with adapted bootstrappes [71], in (C) the 90% and the 95% CI have been plotted. (E) The evolution of the phase of the two time series computed based on wavelet decomposition for the seasonal mode, blue dashed line for the normalized *β*(*t*) red dashed line for the normalized averaged temperature and black dotted line for their phase difference. The graph on the right shows the distribution of the phase differences.
(PDF)

S19 Fig Association between dengue transmission rate and monthly average minimum temperature recorded at the Phnom Penh International Airport (Cambodia). (A) Time evolution of the normalized *β*(*t*) (blue line) and normalized average temperature (red line). (B) and (C) Wavelet Power Spectrum (WPS) [48,70] of the two time series. The graph on the right shows the average WPS. (D) Wavelet coherence [48,70] between the reconstructed *β*(*t*) and average temperature. In (B), (C) and (D) the colors code for low values in white to high values in dark red. The dashed lines show the 95% CI computed with adapted bootstrappes [71], in (C) the 90% and the 95% CI have been plotted. (E) The evolution of the phase of the two time series computed based on wavelet decomposition for the seasonal mode, blue dashed line for the normalized *β*(*t*) red dashed line for the normalized averaged temperature and black dotted line for their phase difference. The graph on the right shows the distribution of the phase differences.
(PDF)

S20 Fig Association between dengue transmission rate and monthly rainfall recorded at the Phnom Penh International Airport (Cambodia). (A) Time evolution of the normalized *β*(*t*) (blue line) and normalized monthly rainfall (red line). (B) and (C) Wavelet Power Spectrum (WPS) [48,70] of the two time series. The graph on the right shows the average WPS. (D) Wavelet coherence [48,70] between the reconstructed *β*(*t*) and monthly rainfall. In (B), (C) and (D) the colors code for low values in white to high values in dark red. The dashed lines show the 95% CI computed with adapted bootstrappes [71], in (C) the 90% and the 95% CI have been plotted. (E) The evolution of the phase of the two time series computed based on wavelet decomposition for the seasonal mode, blue dashed line for the normalized *β*(*t*) red dashed line for the normalized monthly rainfall and black dotted line for their phase difference. The graph on the right shows the distribution of the phase differences.
(PDF)

S21 Fig Association between dengue transmission rate and the Dipole Mode Index (DMI), a proxy of Ocean Indian Dipole (see [72] and http://www.jamstec.go.jp/frcgc/research/d1/iod/HTML/Dipole%20Mode%20Index.html). (A) Time evolution of the normalized *β*(*t*) (blue line) and normalized DMI (red line). (B) and (C) Wavelet Power Spectrum (WPS) [48,70] of the two time series. The graph on the right shows the average WPS. (D) Wavelet coherence [48,70] between the reconstructed *β*(*t*) and DMI. In (B), (C) and (D) the colors code for low values in white to high values in dark red. The dashed lines show the 95% CI computed with adapted bootstrappes [71], in (C) the 90% and the 95% CI have been plotted. (E) The evolution of the phase of the two time series computed based on wavelet decomposition for the seasonal mode, blue dashed line for the normalized *β*(*t*) red dashed line for the normalized DMI and black dotted line for their phase difference. The graph on the right shows the distribution of the phase differences.
(PDF)
