# Correction: Accounting for non-stationarity in epidemiology by embedding time-varying parameters in stochastic models

**DOI:** 10.1371/journal.pcbi.1007062

**Published:** 2019-05-28

**Authors:** The PLOS Computational Biology Staff

There are at least three errors in the manuscript, they are as follows:

- In the “**Models with time-varying parameters**” paragraph after equation (4), for the sentences

“*ε*^*i*^*(t)* describe the clustering of the population [39,40] but can also describe a reduction in the population due to voluntary avoidance behavior or social distancing. However due to the absence of structural identifiability properties [41, 42] it should be very difficult to estimate simultaneously both *β(t)* and *ε*^*i*^*(t)*.”

“*ε*^*i*^(*t*)” must be replaced by “*ε*_*i*_(*t*)” in accordance with the rest of the manuscript. Then these sentences become:

“*ε*_*i*_*(t)* describe the clustering of the population [39,40] but can also describe a reduction in the population due to voluntary avoidance behavior or social distancing. However due to the absence of structural identifiability properties [41, 42] it should be very difficult to estimate simultaneously both *β(t)* and *ε*_*i*_*(t)*.”

- In the Fig 3 caption the equation of *β*(*t*) must be read as:

“*β*(t) = *β*_*0*_.(1 + *β*_*1*_ sin(2*πt*/365+2*πϕ*) + *β*_*2*_ sin(2*πt*/(3 365)+2*πϕ*) + *β*_3_ sin(2*πt*/(0.5 365)+2*πϕ*))

and not:

“*β*(t) = *β*_*0*_.(1 + *β*_*1*_ sin(2*πt*/365+2*πϕ*) + *β*_*2*_ sin(2*πt*/(3 365)+2*πϕ*)) + *β*_3_ sin(2*πt*/(0.5 365)+2*πϕ*))

- In the “**SIRS model**” paragraph after equation (5), the sentence

“Initially non-informative priors were used for the volatility *σ*, the reporting rate ρ and the initial conditions *γ*(0) by *β*(0) but to reduce problems linked to practical non-identifiability materialized by correlation between some estimates, informative priors were used for ρ (see S1 Fig).”

must be replaced (as in the uncorrected proofs) by:

“Initially non-informative priors were used for the volatility *σ*, the reporting rate ρ and the initial conditions (*S*(0), *I*(0), *β*(0)) to reduce problems linked to practical non-identifiability materialized by correlation between some estimates, informative priors were used for ρ (see S1 Fig).”
