# Supplementary material for: Accounting for non-stationarity in epidemiology by embedding time-varying parameters in stochastic models
Source: PLoS Comput Biol. 2018 Aug 15;14(8):e1006211. doi: 10.1371/journal.pcbi.1006211 (PMC6110518; doi:10.1371/journal.pcbi.1006211)
Supplement: S1 Table — The test was implemented with the Coda package in R [66]. (PDF) [file pcbi.1006211.s002.pdf]

Table S1. Test of the MCMC chains: Geweke diagnosis [67] that tests for the non-stationarity of the chains, the parameter means computed using the first 10% and the last 50% of the chain are compared through a Z-score (stationarity is not rejected if the Z-scores are below the critical values at 5%, **NS** (non-significant) in the Table). The test was implemented with the Coda package in R [66].

**Parameter**	**Fig. 1**	**Fig. 3A**	**Fig. 3B**	**Fig. 3C**	**Fig. 4**	**Fig. 5**	**Fig. 6**	**Fig. S4**	**Fig. S7**	**Fig. S9**	**Fig. S11**
$\beta(0)$	**NS**	–	–	–	–	**NS**	**NS**	–	–	–	–
$S(0)$	**NS**	**NS**	**NS**	–	**NS**	**NS**	**NS**	**NS**	**NS**	–	**NS**
$E(0)$	–	–	–	–	–	–	**NS**	–	–	–	–
$I(0)$	**NS**	**NS**	**NS**	–	**NS**	**NS**	–	**NS**	**NS**	–	**NS**
i	–	–	–	–	–	**NS**	**NS**	–	–	–	–
γ	**NS**	**NS**	**NS**	–	**NS**	**NS**	**NS**	**NS**	**NS**	–	**NS**
α	**NS**	**NS**	**S**	–	**NS**	**NS**	**NS**	**NS**	**NS**	–	**NS**
β_0	–	–	–	–	–	–	–	–	–	–	**NS**
β_1	–	–	–	–	–	–	–	–	–	–	**NS**
ϕ	–	–	–	–	–	–	–	–	–	–	**NS**
ρ	**NS**	–	–	–	–	–	**NS**	–	–	–	**NS**
σ	**NS**	**NS**	**NS**	**NS**	**NS**	**NS**	**NS**	**NS**	**NS**	**NS**	–
