# Supplementary figures and images for: Accounting for non-stationarity in epidemiology by embedding time-varying parameters in stochastic models
Source: PLoS Comput Biol. 2018 Aug 15;14(8):e1006211. doi: 10.1371/journal.pcbi.1006211 (PMC6110518; doi:10.1371/journal.pcbi.1006211)

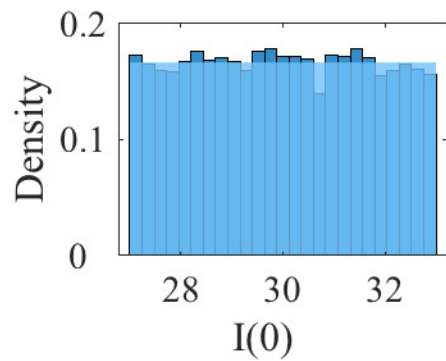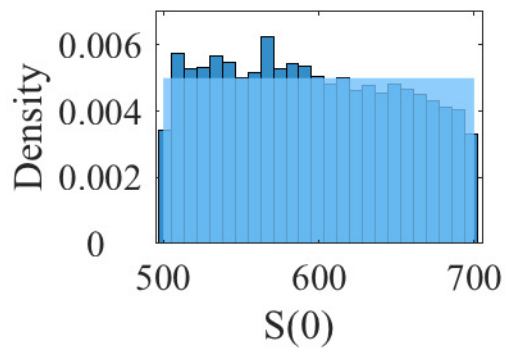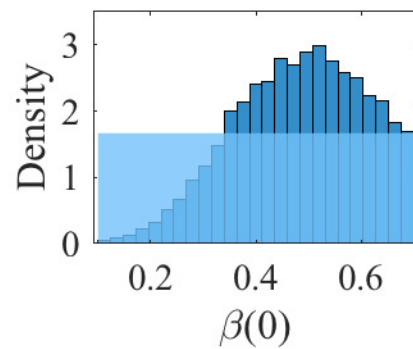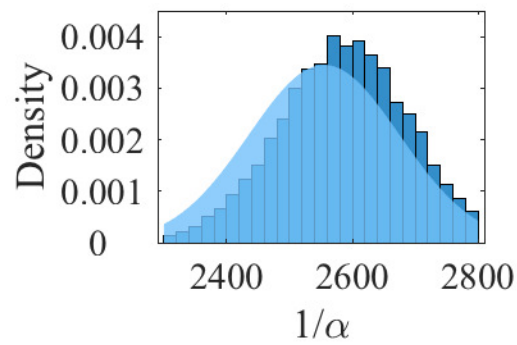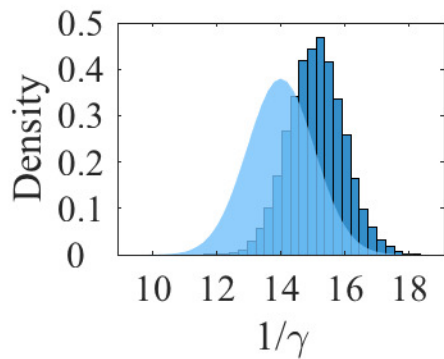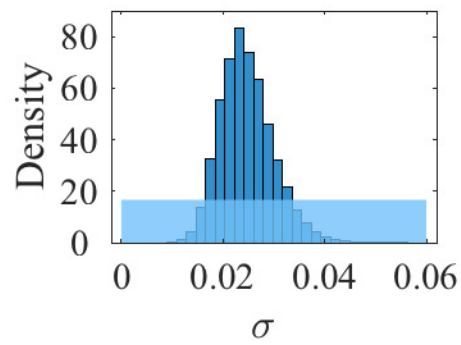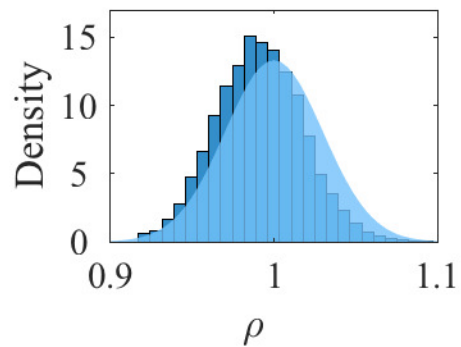

Supplement: S1 Fig — I(0), S(0) initial values, β(0) initial value of β(t), 1/α is the average duration of immunity, γ is the recovery rate, ρ is the reporting rate and σ is the volatility of the Brownian process of β(t). The blue distributions are the priors and the discrete histograms are the posteriors. The medians of the prior distributions for I(0), S(0), β(0), 1/α, 1/γ and ρ are the “true values” used for the simulations of the observed incidences. (PDF) [file pcbi.1006211.s004.pdf]

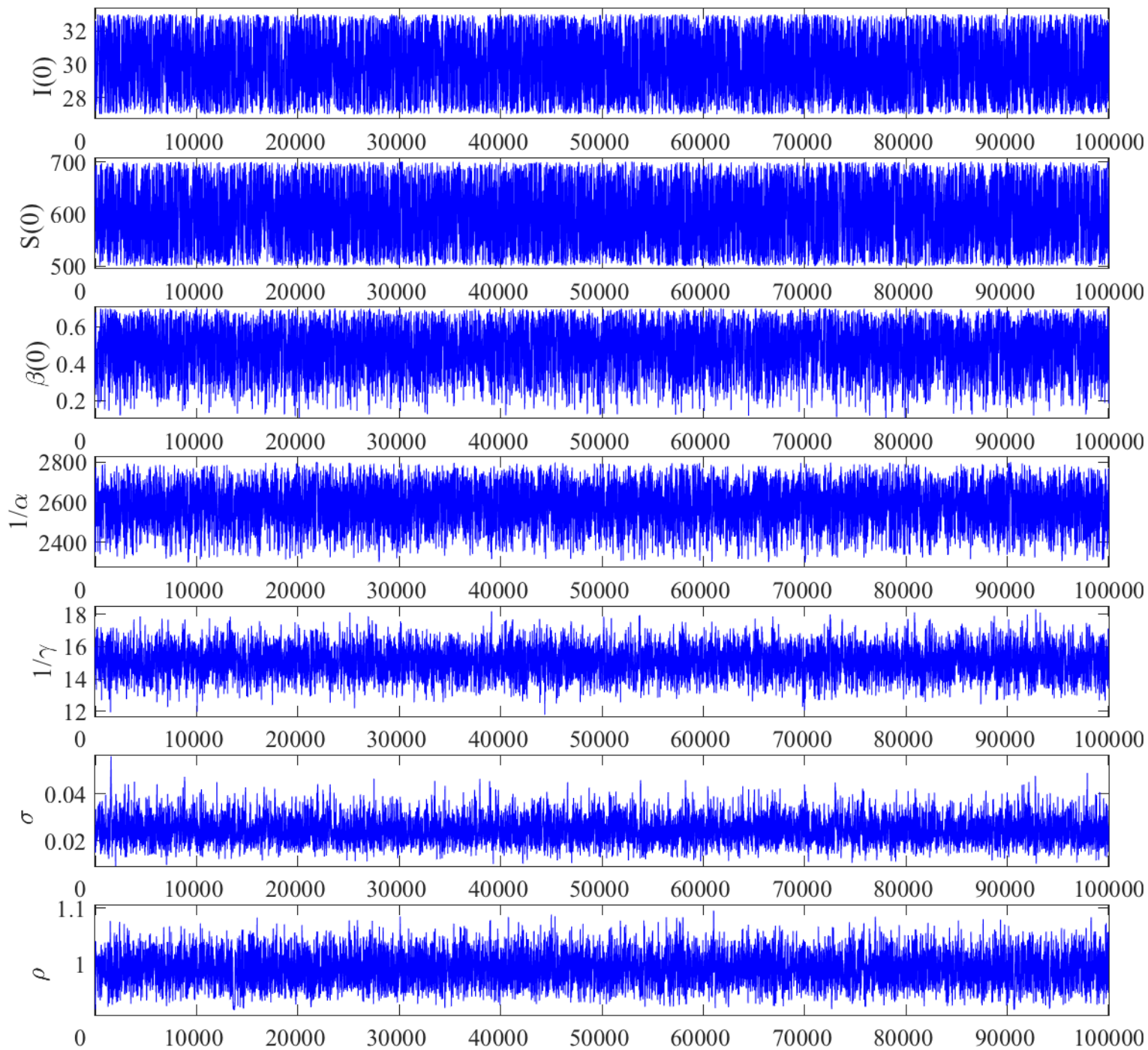

Supplement: S2 Fig — I(0), S(0) initial values, β(0) initial value of β(t), 1/α is the average duration of immunity, γ is the recovery rate, ρ is the reporting rate and σ is the volatility of the Brownian process of β(t). (PDF) [file pcbi.1006211.s005.pdf]

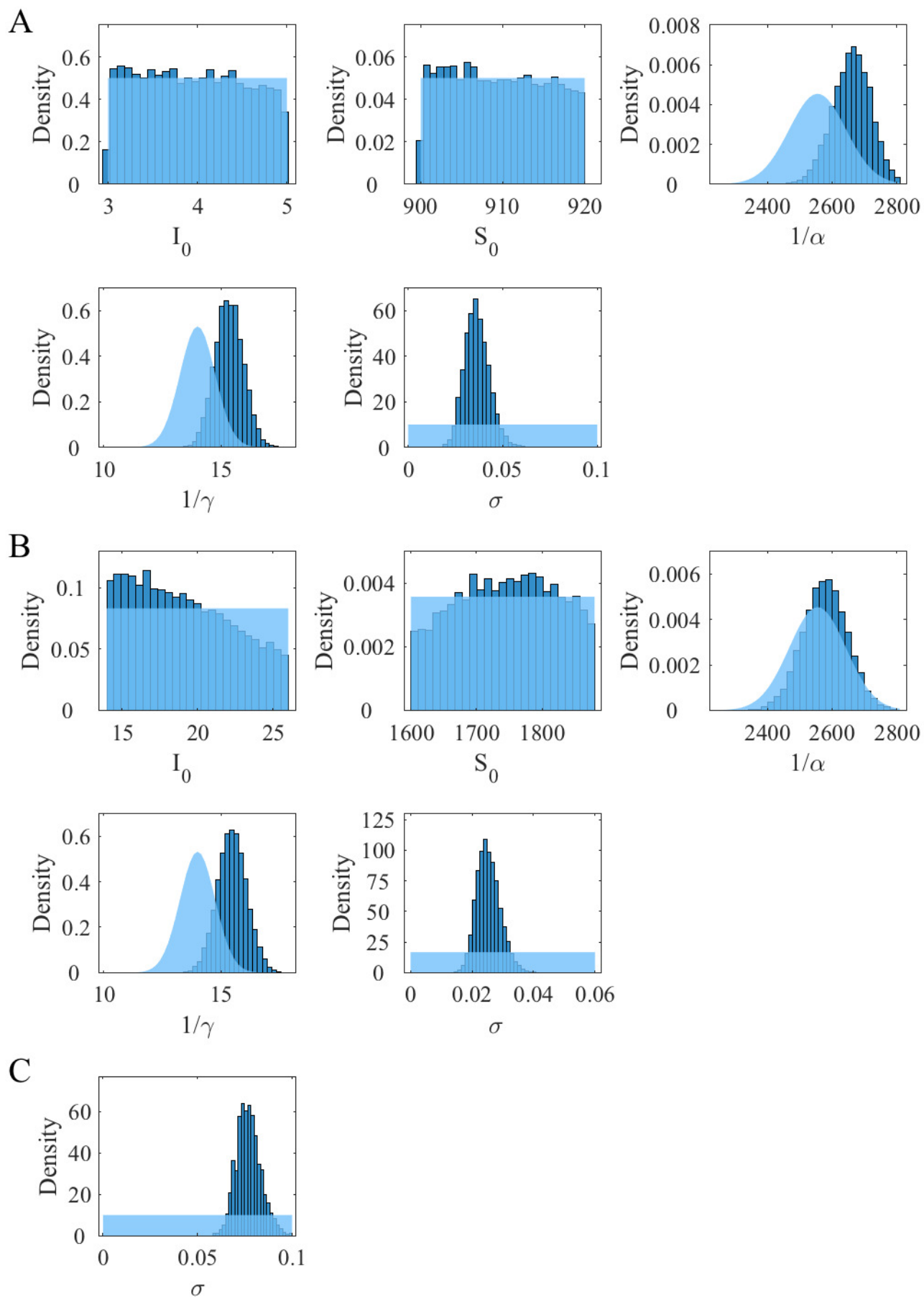

Supplement: S3 Fig — A/ Observed data generated with a SIRS model and a sinusoidal β with 1 periodic component (5). B/ Observed data generated with a SIRS model and a sinusoidal β with 2 periodic components. In A/ and B/, I(0), S(0) are initial values, 1/α is the average duration of immunity, γ is the recovery rate and σ is the volatility of the Brownian process of β(t). C/ Observed data generated with a SIRS model and a sinusoidal β with 3 periodic components, σ the volatility of the Brownian process of β(t) is the only parameter inferred. The blue distributions are the priors and the discrete histograms are the posteriors. The medians of the prior distributions are the “true values” used for the simulations of the observed incidences. (PDF) [file pcbi.1006211.s006.pdf]

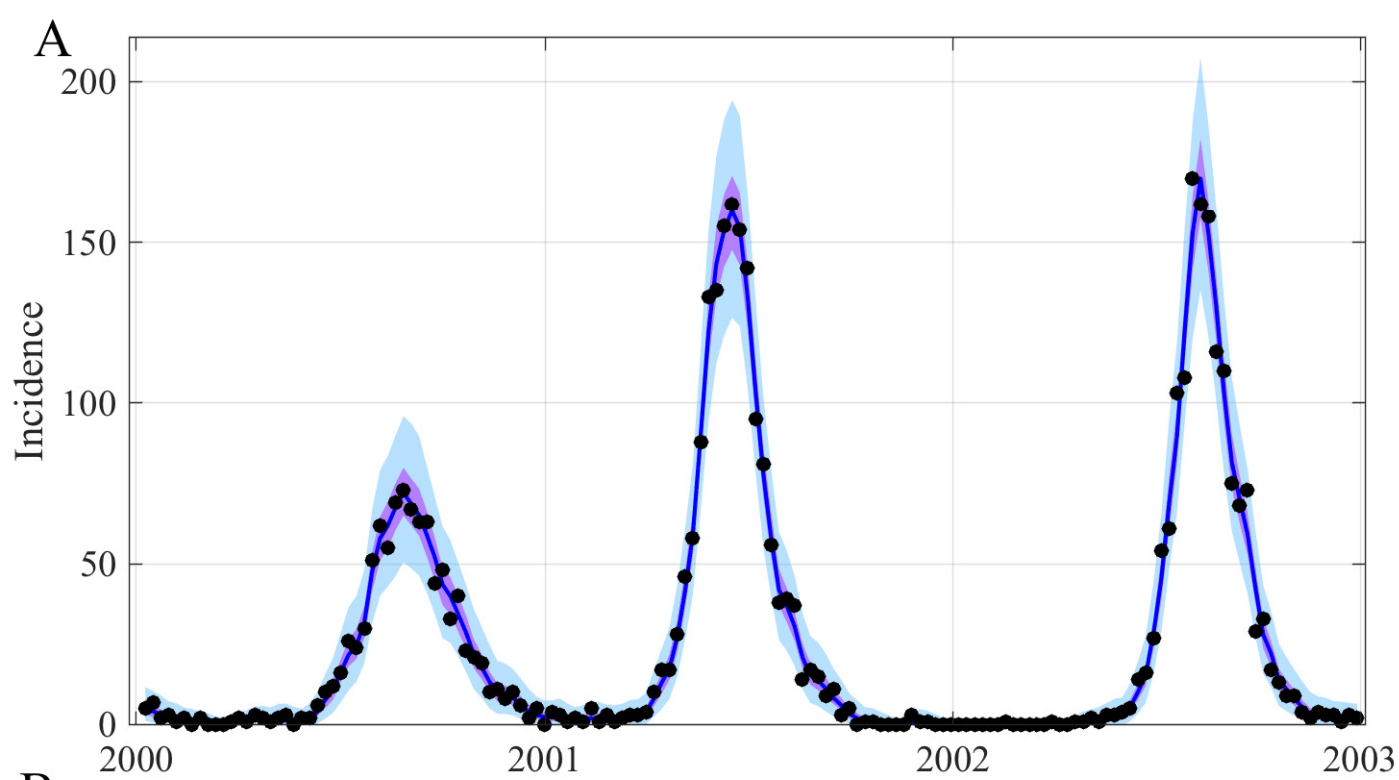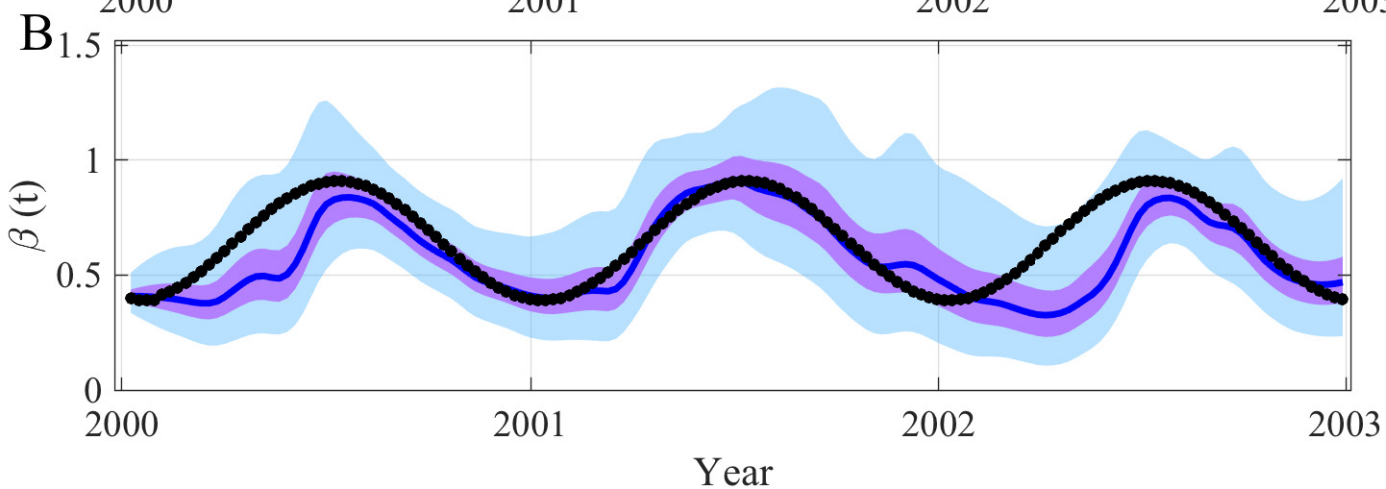

Supplement: S4 Fig — Reconstruction of both the incidence (A) and the time evolution β(t) (B) for the SIRS model as in Fig 1 but only 5 parameters have been inferred, β(0) and ρ were fixed. Model parameters as in Fig 1 and S6 Fig. (PDF) [file pcbi.1006211.s007.pdf]

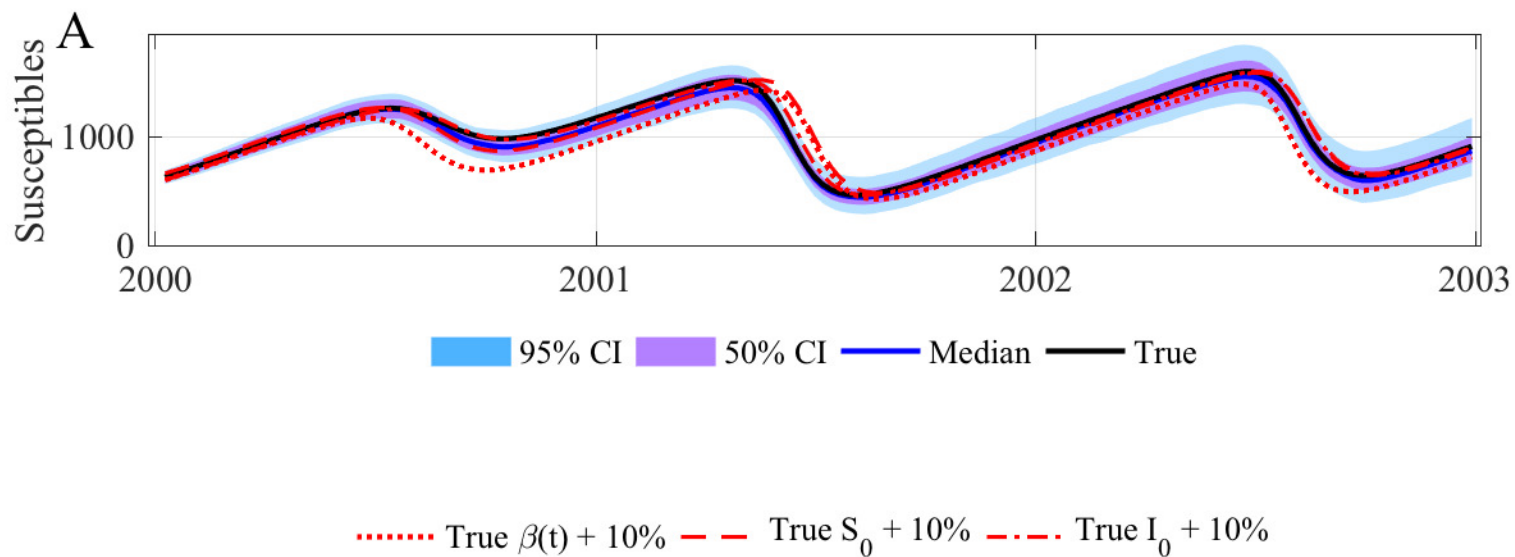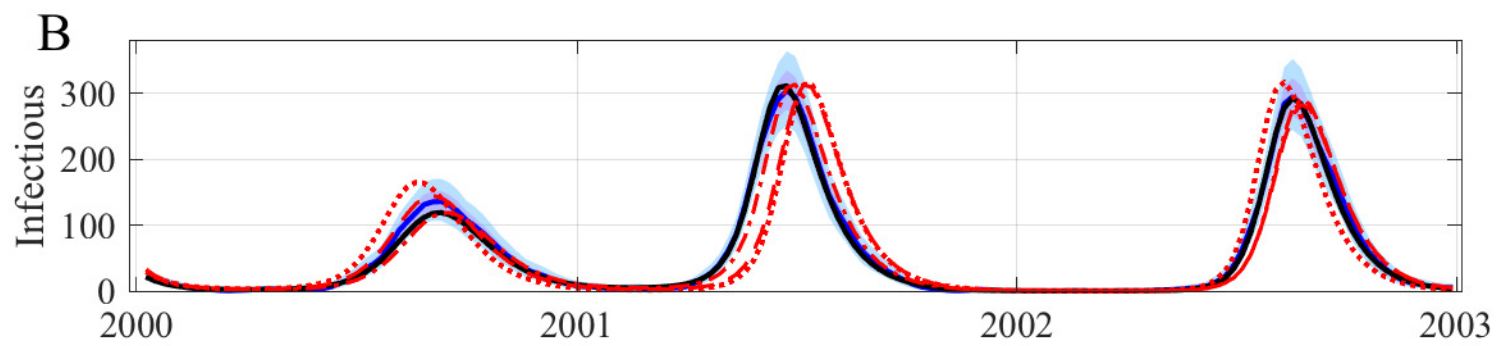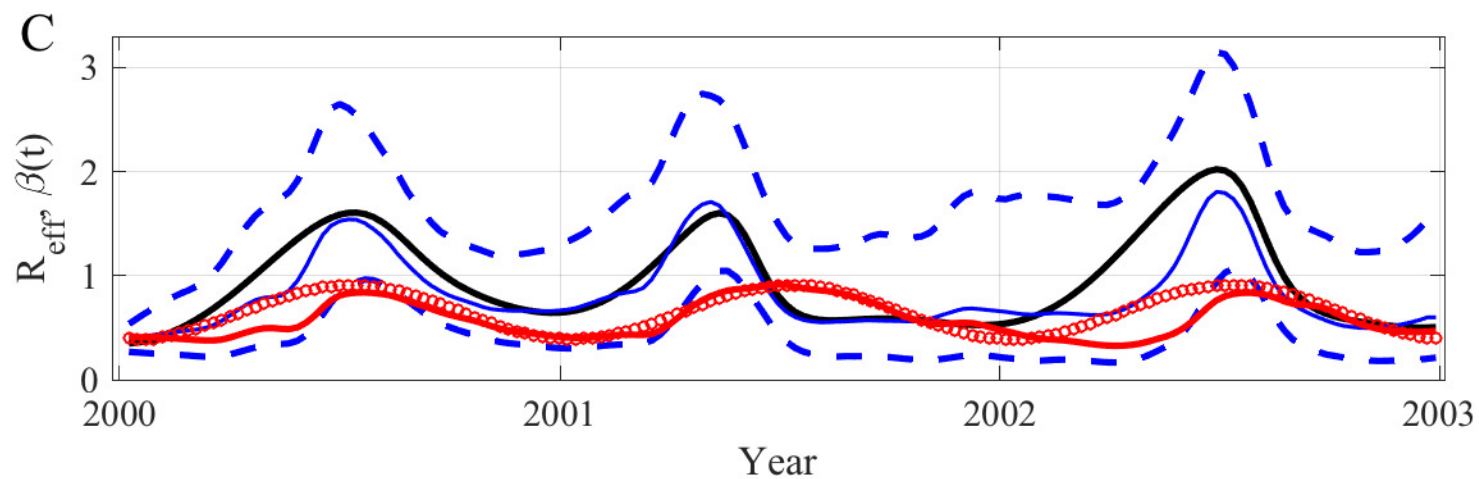

Supplement: S5 Fig — Simulation of the SIRS model when the initial conditions are near the attractor of the dynamics and just 5 parameters inferred: (A) Susceptibles; (B) Infectious; (C) Time evolution of both Reff and β(t). In (A) and (B) the black lines are the true values, the blue lines are the median of the posterior, the mauve areas are the 50% CI and the light blue areas the 95% CI. In (C) the black line is the true values of Reff, the blue line is the median of the posterior, and the dashed lines the 95% CI of Reff; the red dot line is the true time evolution of β(t) and the red line the median of its posterior. Model parameters as in Fig 1 and S6 Fig. (PDF) [file pcbi.1006211.s008.pdf]

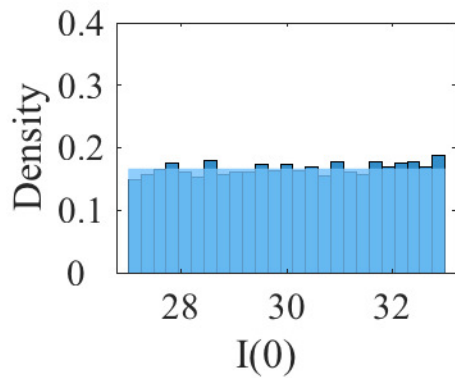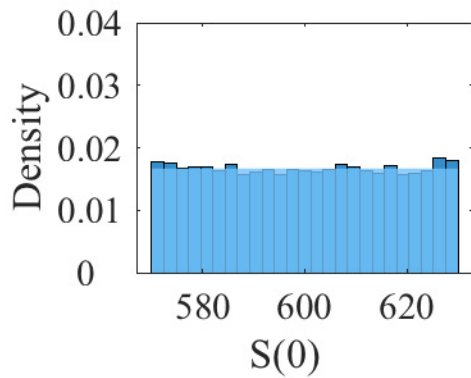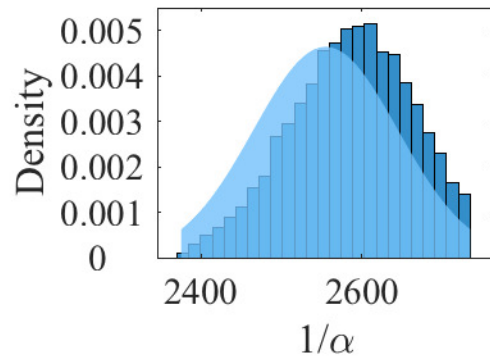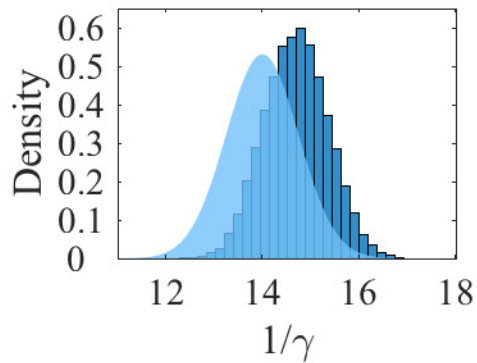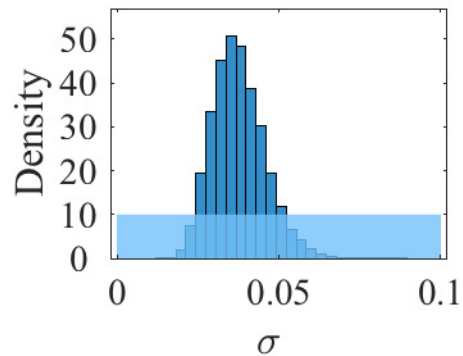

Supplement: S6 Fig — I(0), S(0) initial values, 1/α is the average duration of immunity, γ is the recovery rate and σ is the volatility of the Brownian process of β(t). The blue distributions are the priors and the discrete histograms are the posteriors. The medians of the prior distributions for I(0), S(0), 1/α and 1/γ, are the “true values” used for the simulations of the observed incidences. (PDF) [file pcbi.1006211.s009.pdf]

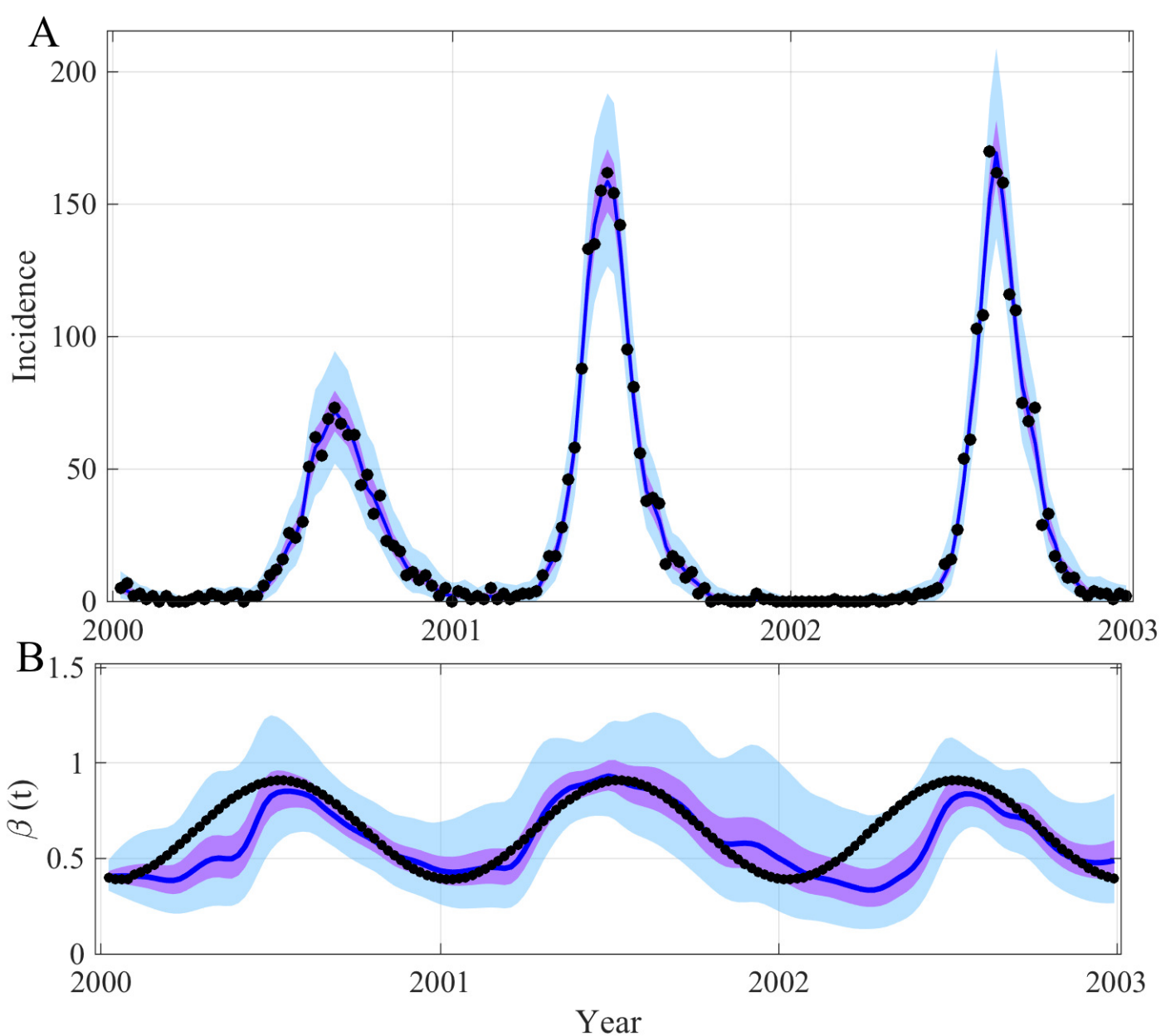

Supplement: S7 Fig — Reconstruction of both the incidence (A) and the time evolution β(t) (B) for the SIRS model as in Fig 1 but σ the volatility of the Brownian process of β(t) is the only parameter inferred. Model parameters as in Fig 1 and S8 Fig. (PDF) [file pcbi.1006211.s010.pdf]

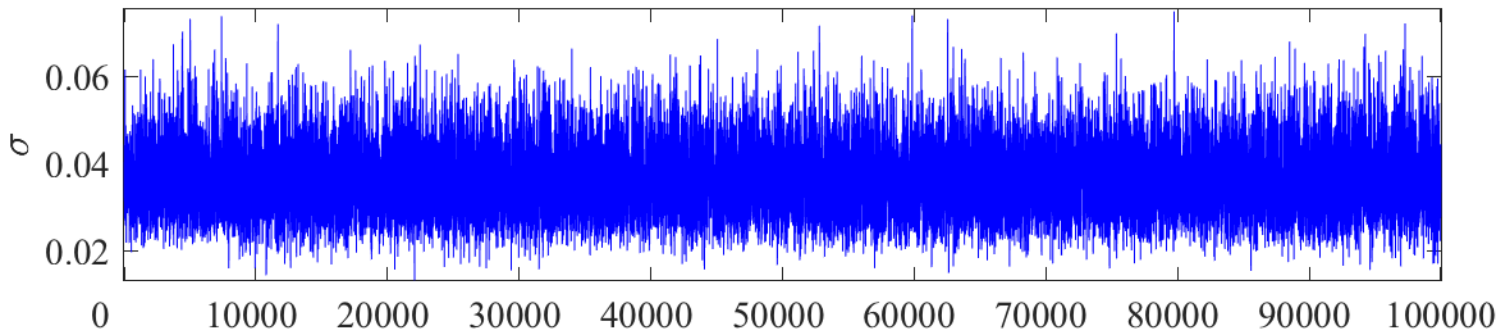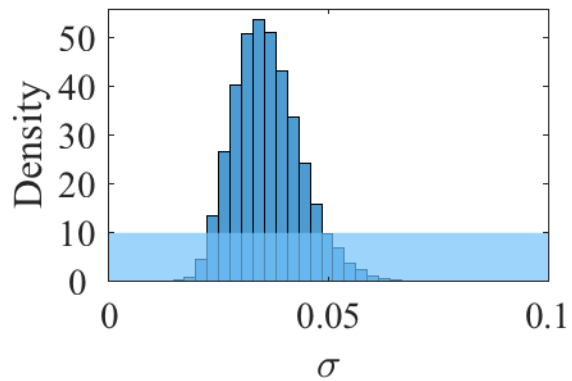

Supplement: S8 Fig — (PDF) [file pcbi.1006211.s011.pdf]

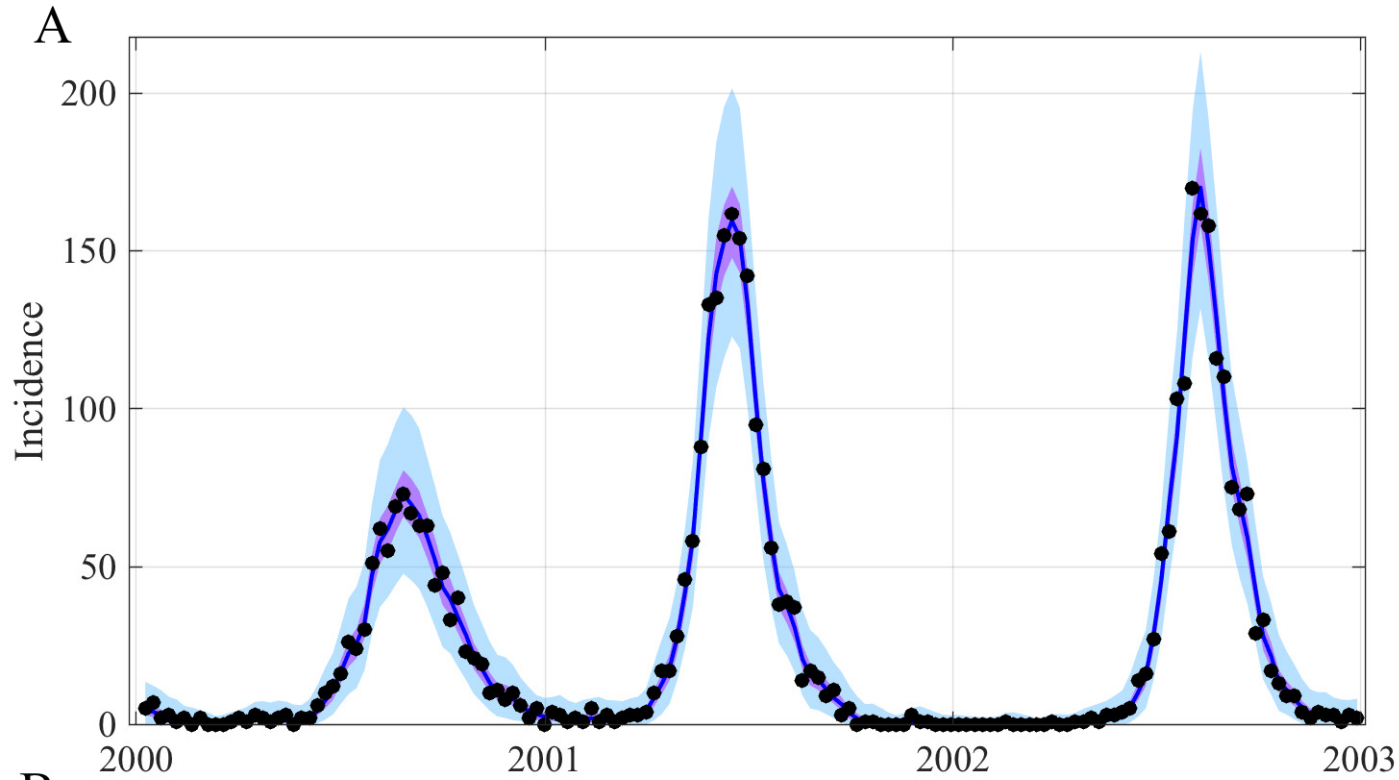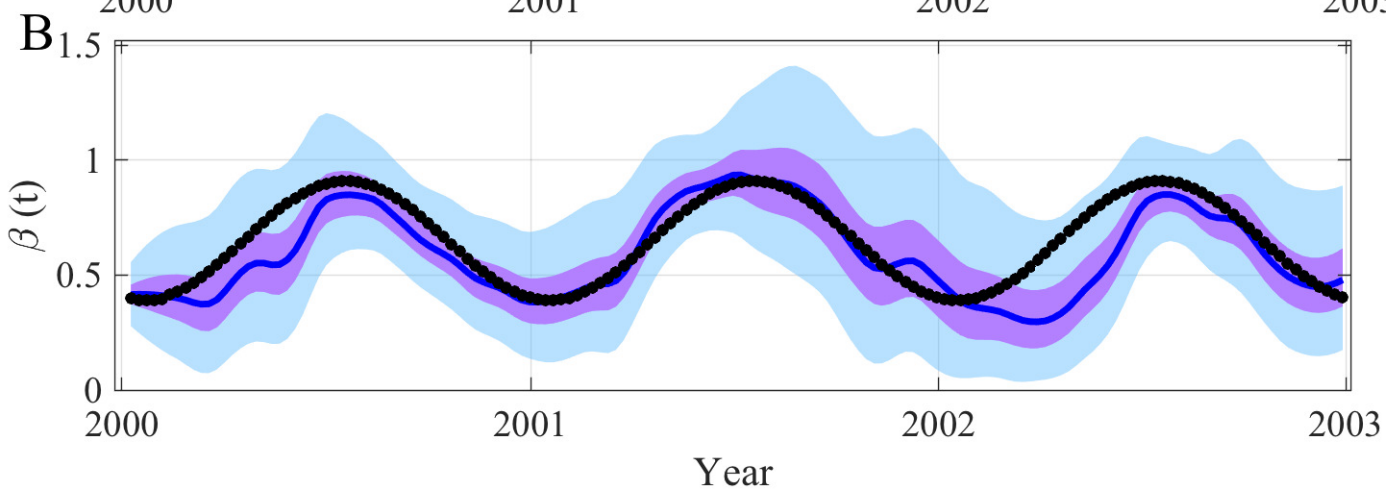

Supplement: S9 Fig — Reconstruction of both the incidence (A) and the time evolution β(t) (B) for the SIRS model as in S4 Fig but the logarithm transformation of the Brownian process of β(t) is not used: dθ(t) = σ.dB(t). Model parameters as in Fig 1 and S4 Fig. (PDF) [file pcbi.1006211.s012.pdf]

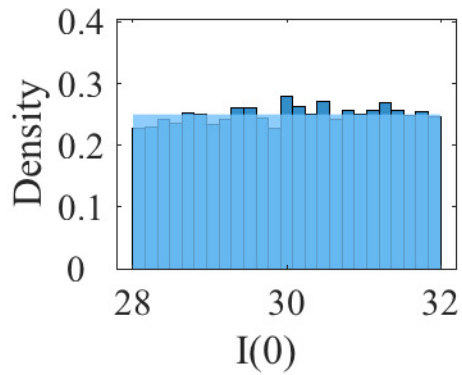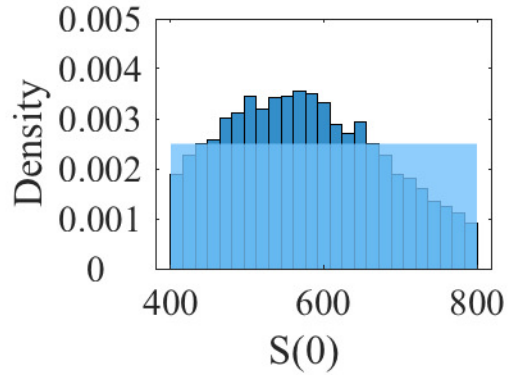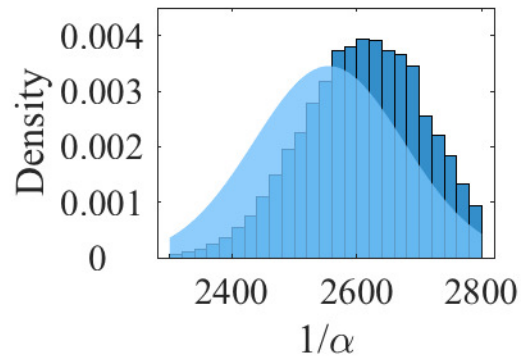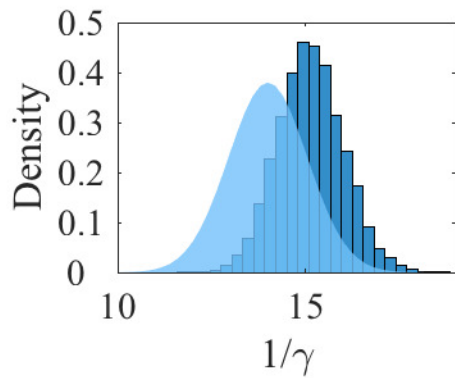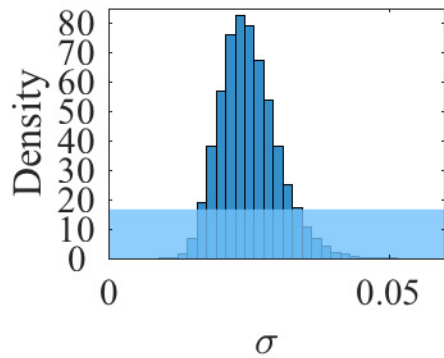

Supplement: S10 Fig — (PDF) [file pcbi.1006211.s013.pdf]

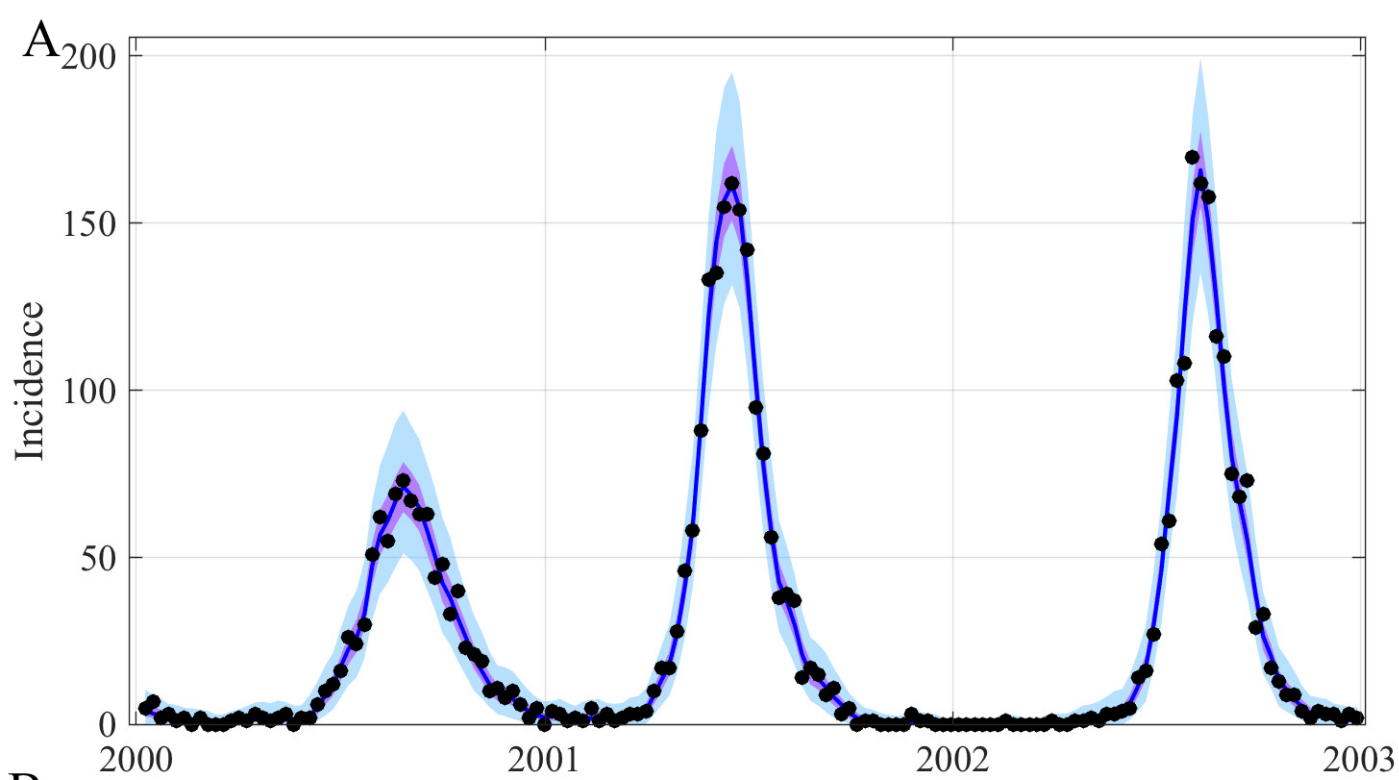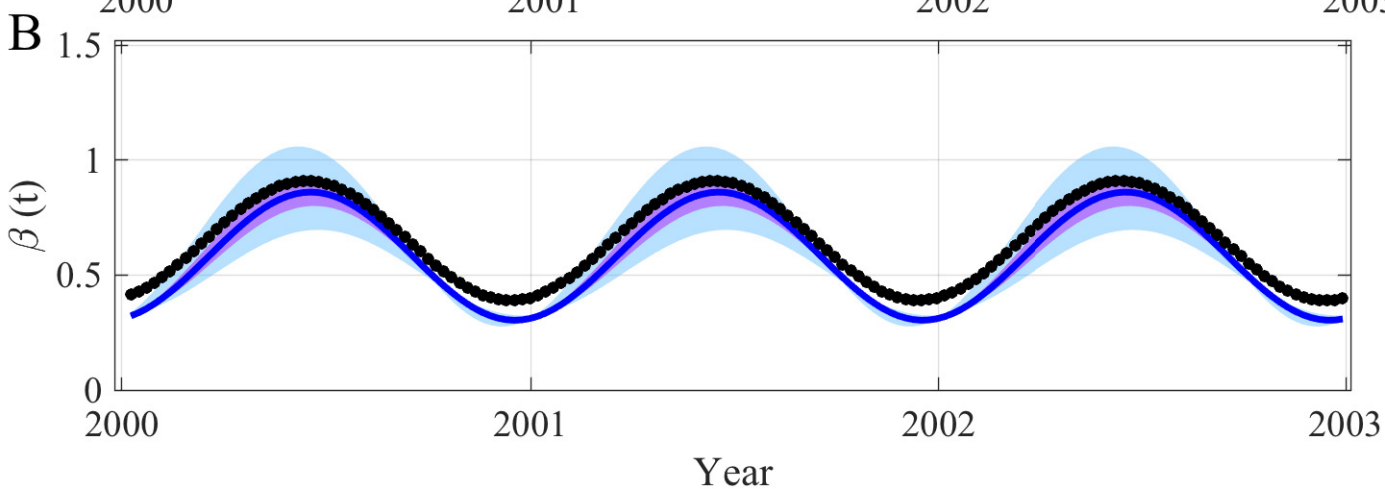

Supplement: S11 Fig — Reconstruction of both the incidence (A) and the time evolution β(t) (B) with the true SIRS model. In (A) the black points are observations generated with a Poisson process with a mean equal to the incidence simulated by the model. In (B) the black points are the true values of β(t) = β0.(1 + β1 sin(2πt/365+2πϕ)). The blue lines are the median of the posterior, the mauve areas are the 50% Credible Intervals (CI) and the light blue areas the 95% CI. For all the figures, the observation process is also applied to the inferred incidence trajectory. The time unit of the model is day, the initial date is arbitrary (2000-01-09) and parameters used for the SIRS model are as follows: μ = 1/(50*365), α = 1/(7*365), γ = 1/14, β0 = 0.65, β1 = 0.4, ϕ = -0.2, ρ = 1, N = 10000, S(0) = 600, I(0) = 30. The prior and posterior distributions of the inferred parameters are in S13 Fig (PDF) [file pcbi.1006211.s014.pdf]

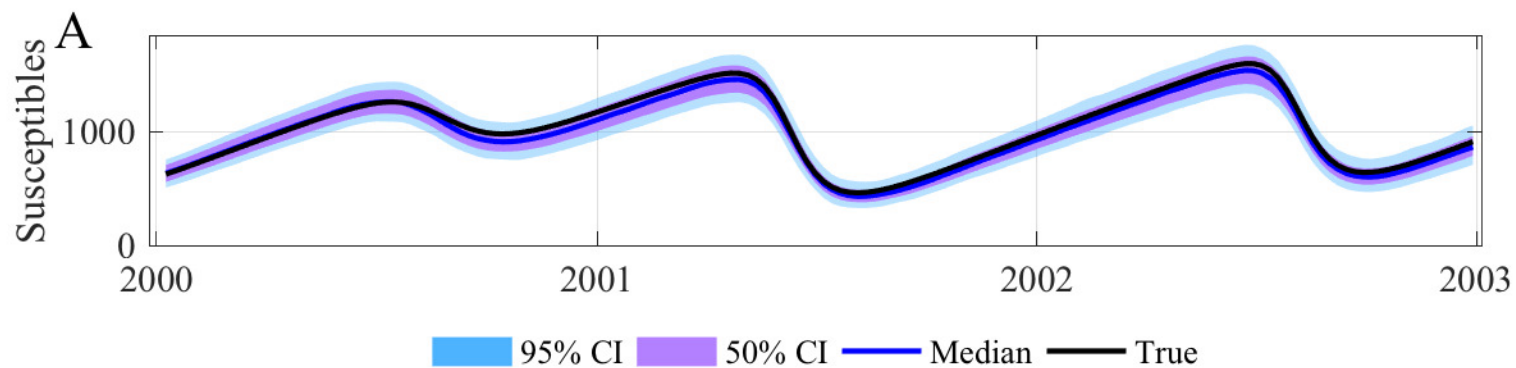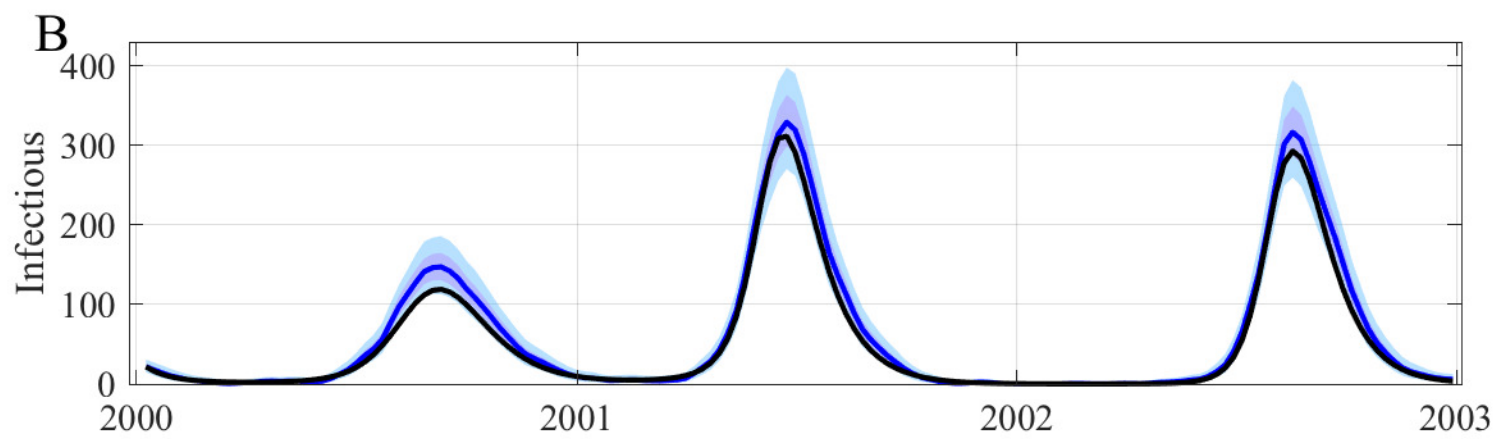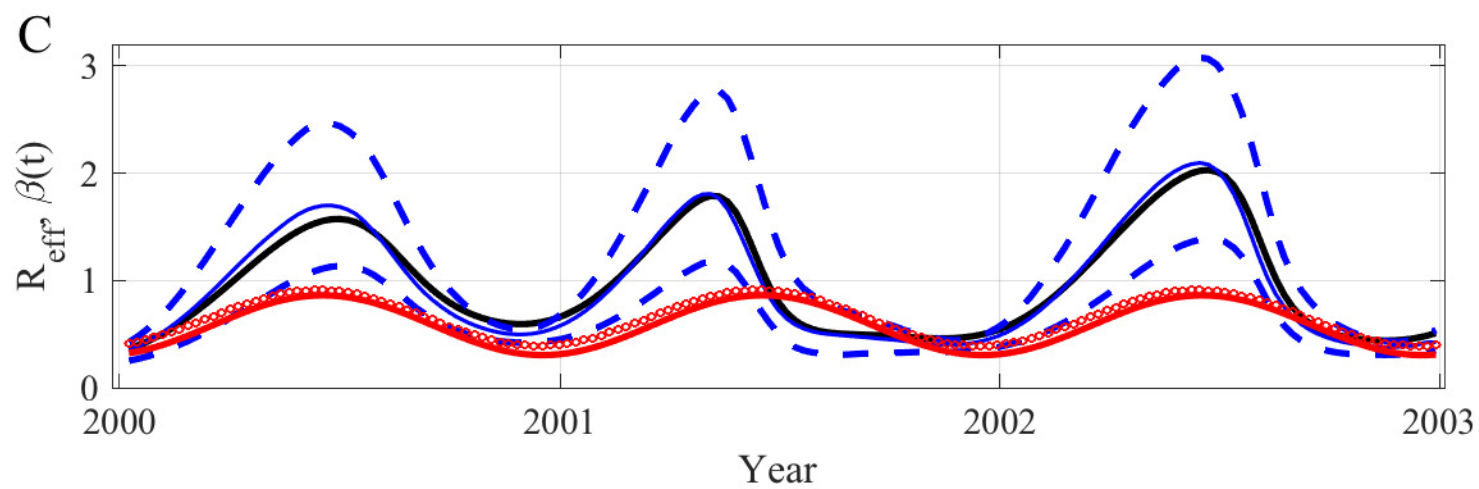

Supplement: S12 Fig — Simulation of the true SIRS model: (A) Susceptibles; (B) Infectious; (C) Time evolution of both Reff and β(t). In (A) and (B) the black lines are the true values, the blue lines are the median of the posterior, the mauve areas are the 50% CI and the light blue areas the 95% CI. In (C) the black line is the true values of Reff, the blue line is the median of the posterior, and the dashed lines the 95% CI of Reff; the red dot line is the true time evolution of β(t) and the red line the median of its posterior. Model parameters as in S11 Fig and S13 Fig. (PDF) [file pcbi.1006211.s015.pdf]

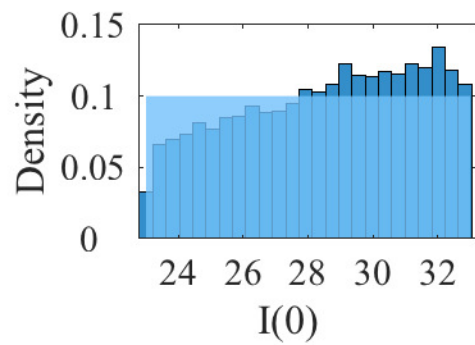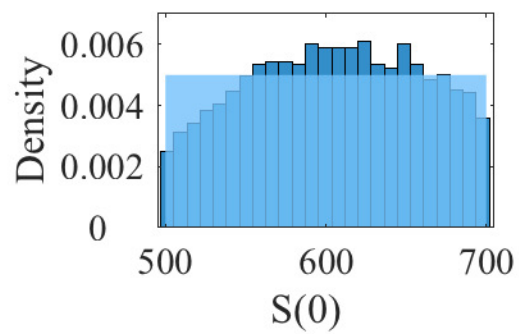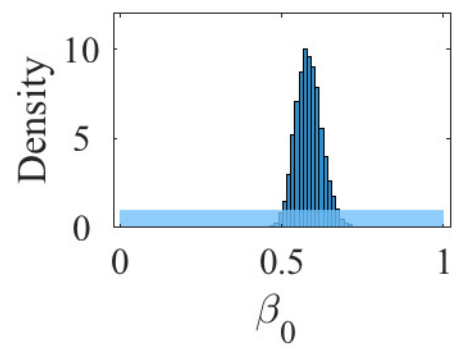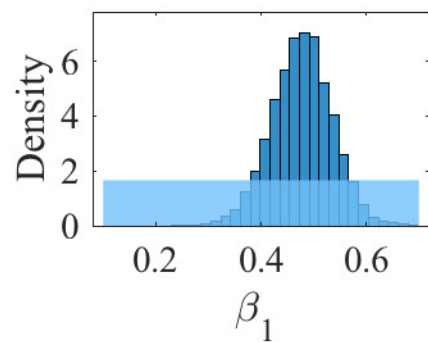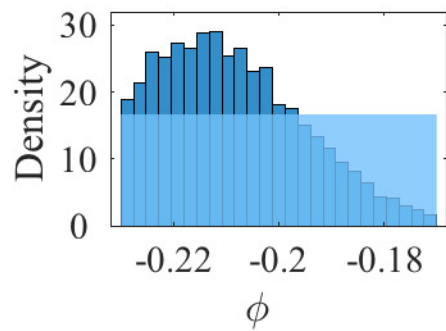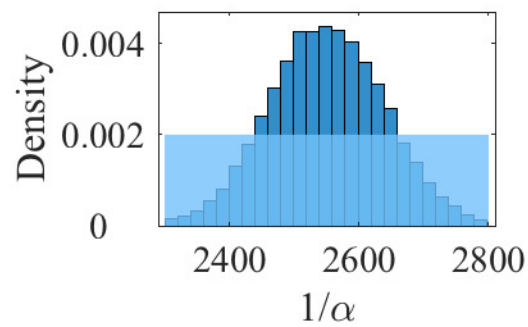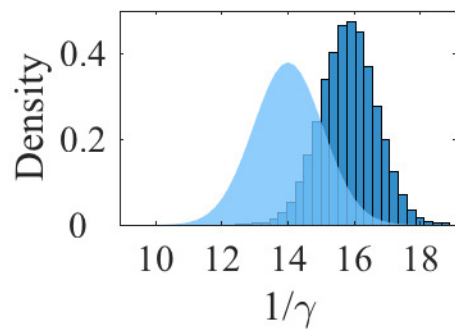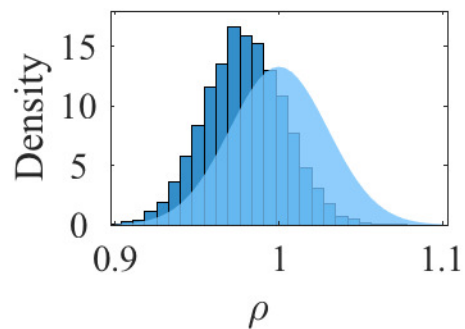

Supplement: S13 Fig — I(0), S(0) initial values, β0, β1, ϕ, the parameters of the sinusoidal β, 1/α is the average duration of immunity, γ is the recovery rate and ρ is the reporting rate. The blue distributions are the priors and the discrete histograms are the posteriors. The medians of the prior distributions are the “true values” used for the simulations of the observed incidences. (PDF) [file pcbi.1006211.s016.pdf]

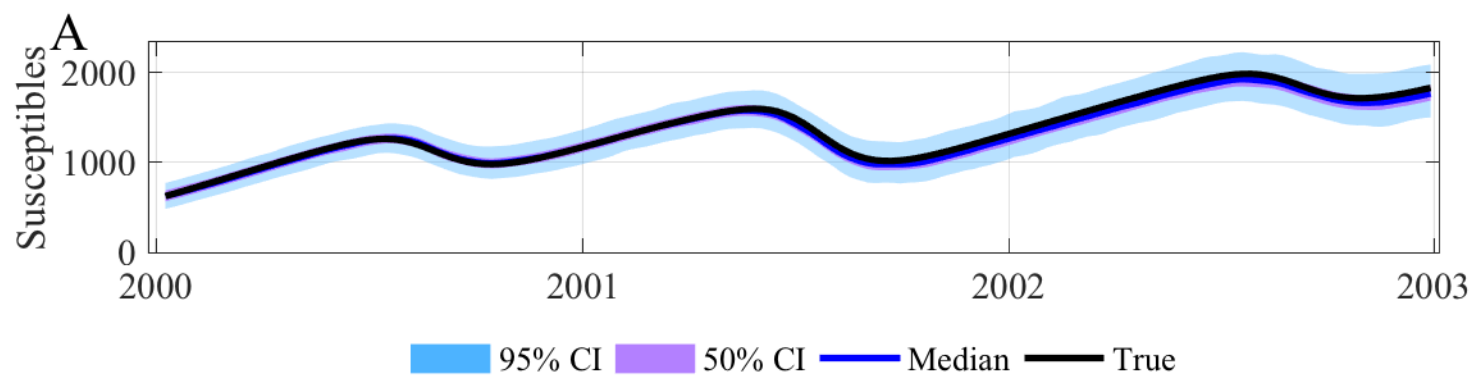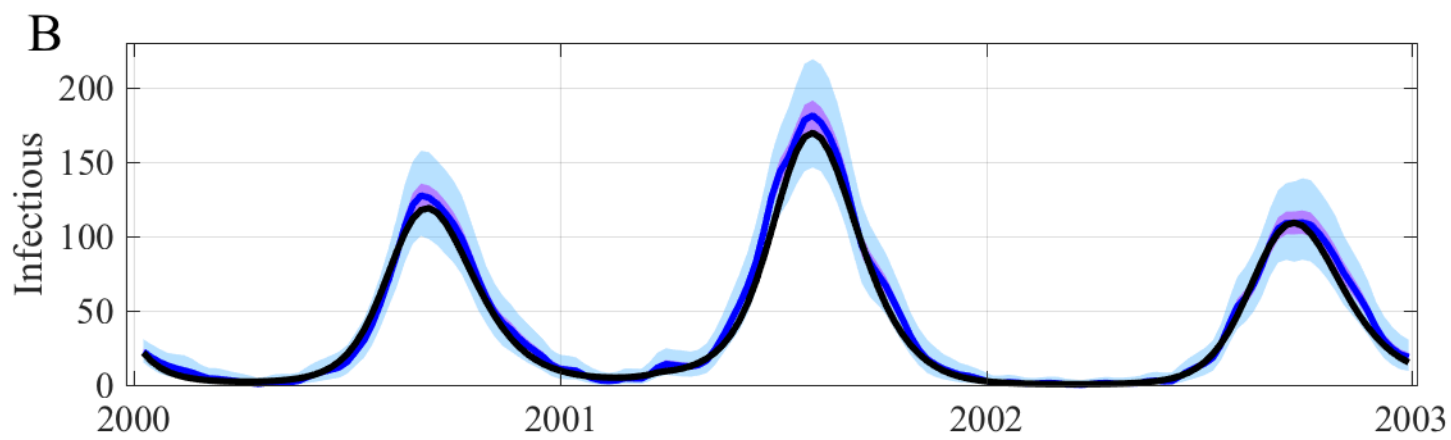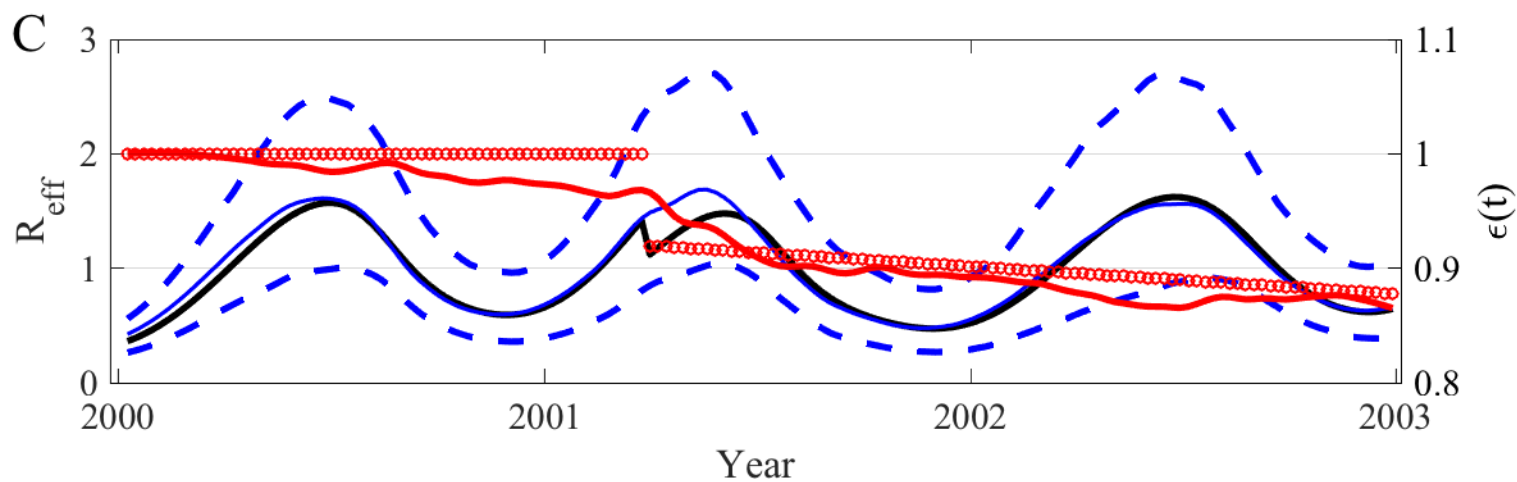

Supplement: S14 Fig — Simulation of the SIRS model: (A) Susceptibles; (B) Infectious; (C) Time evolution of both Reff and εS(t). In (A) and (B) the black lines are the true values, the blue lines are the median of the posterior, the mauve areas are the 50% CI and the light blue areas the 95% CI. In (C) the black line is the true values of Reff, the blue line is the median of the posterior and the dashed lines the 95% CI of Reff; the red dot line is the true time evolution of εS(t) and the red line the median of its posterior. Model parameters as in Fig 4 and S15 Fig. (PDF) [file pcbi.1006211.s017.pdf]

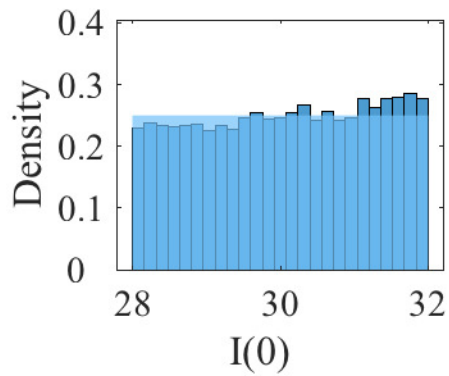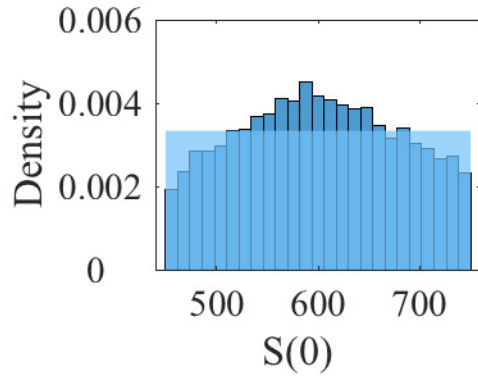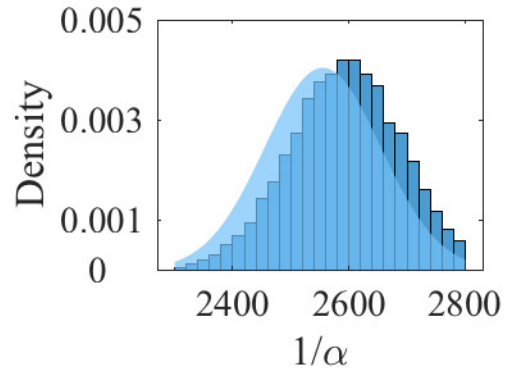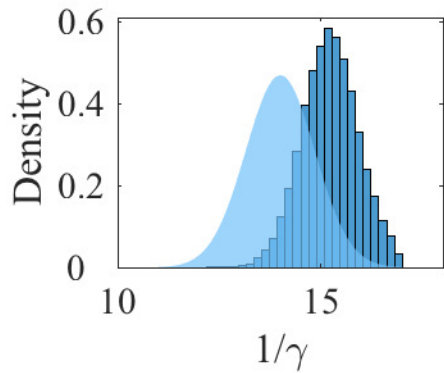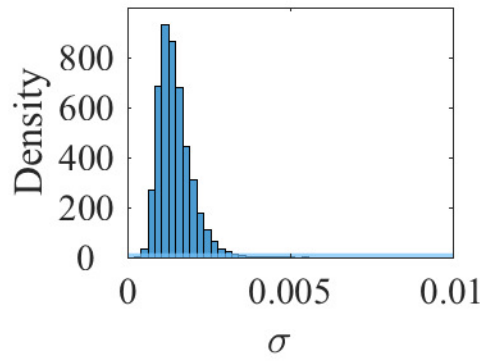

Supplement: S15 Fig — I(0), S(0) initial values, 1/α is the average duration of immunity, γ is the recovery rate and σ is the volatility of the Brownian process of εS(t). The blue distributions are the priors and the discrete histograms are the posteriors. The medians of the prior distributions for I(0), S(0), 1/α and 1/γ, are the “true values” used for the simulations of the observed incidences. (PDF) [file pcbi.1006211.s018.pdf]

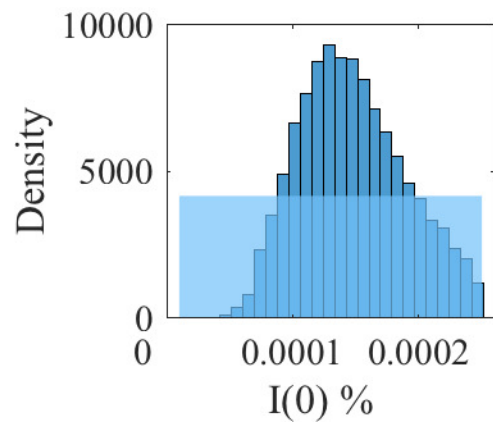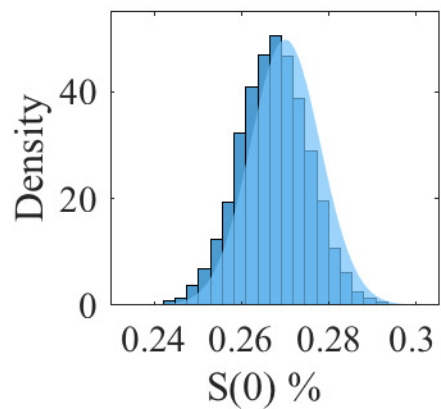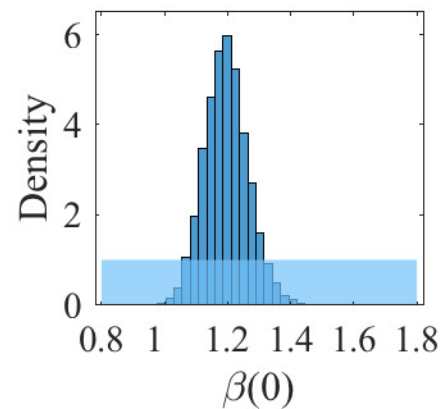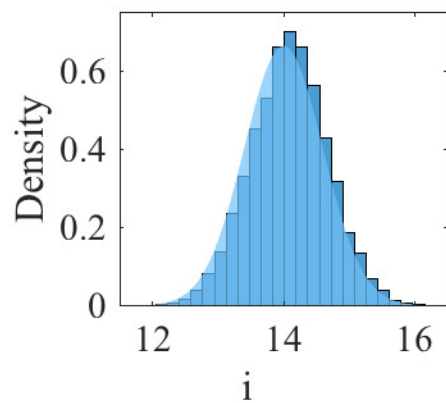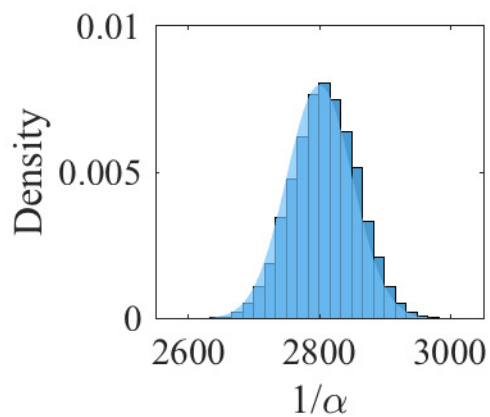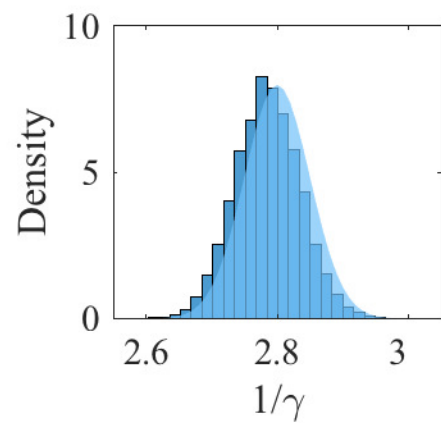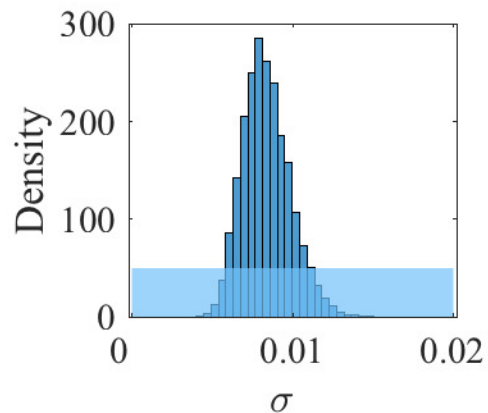

Supplement: S16 Fig — I(0), S(0) initial values expressed in percentage of the population N, β(0) initial value of β(t), i imported infectious, 1/α is the average duration of immunity, γ is the recovery rate and σ is the volatility of the Brownian process of β(t). The blue distributions are the priors and the discrete histograms are the posteriors. Prior values are adapted from [46]. (PDF) [file pcbi.1006211.s019.pdf]

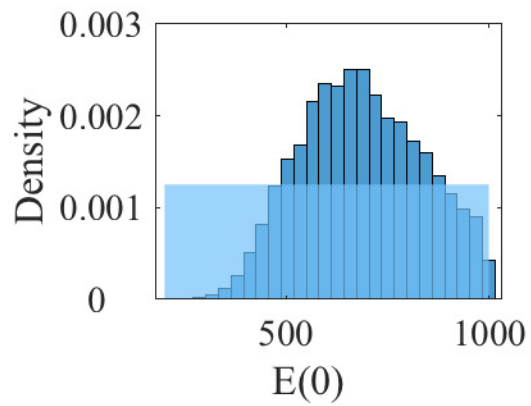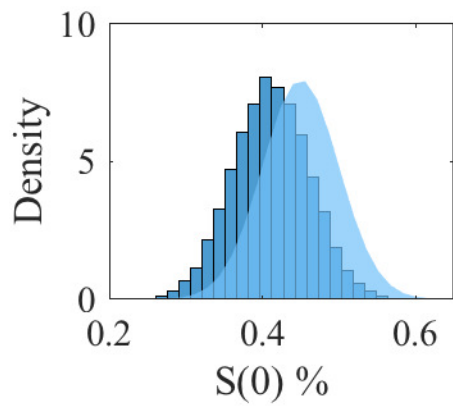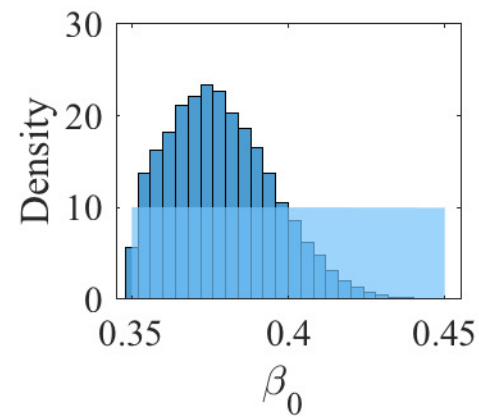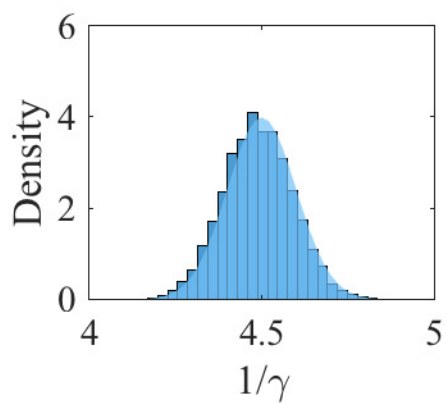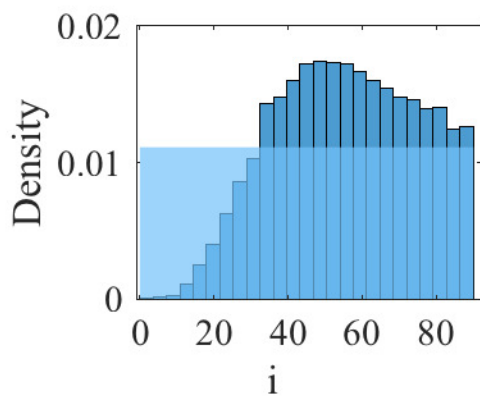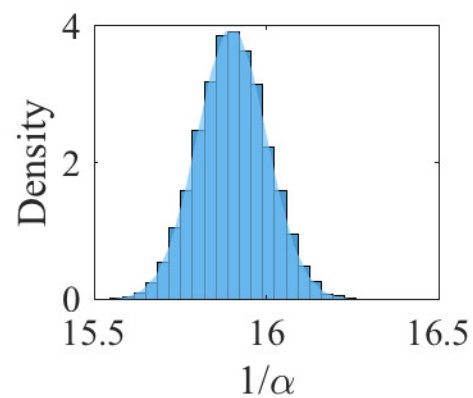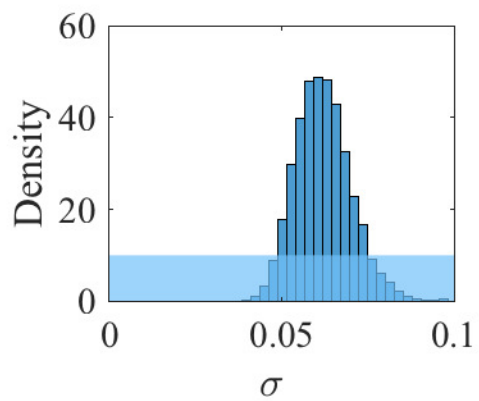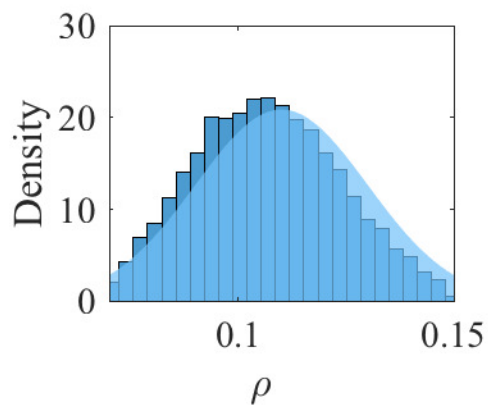

Supplement: S17 Fig — E(0), S(0) expressed in percentage of the population N, β(0) the initial value of β(t), γ is the recovery rate, i imported infectious, 1/α is the average duration of immunity, σ is the volatility of the Brownian process of β(t) and ρ the reporting rate. The blue distributions are the priors and the discrete histograms are the posteriors. Prior values are adapted from [63]. (PDF) [file pcbi.1006211.s020.pdf]

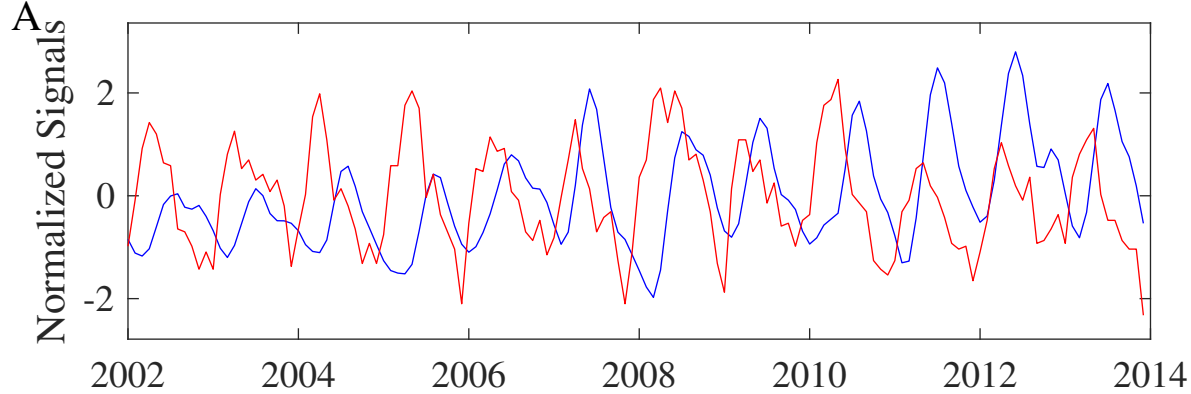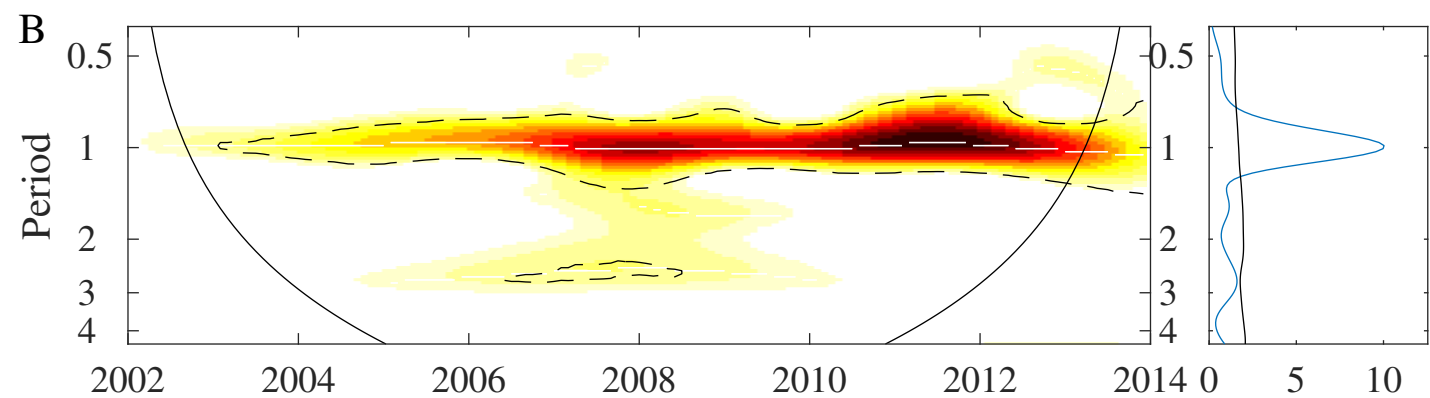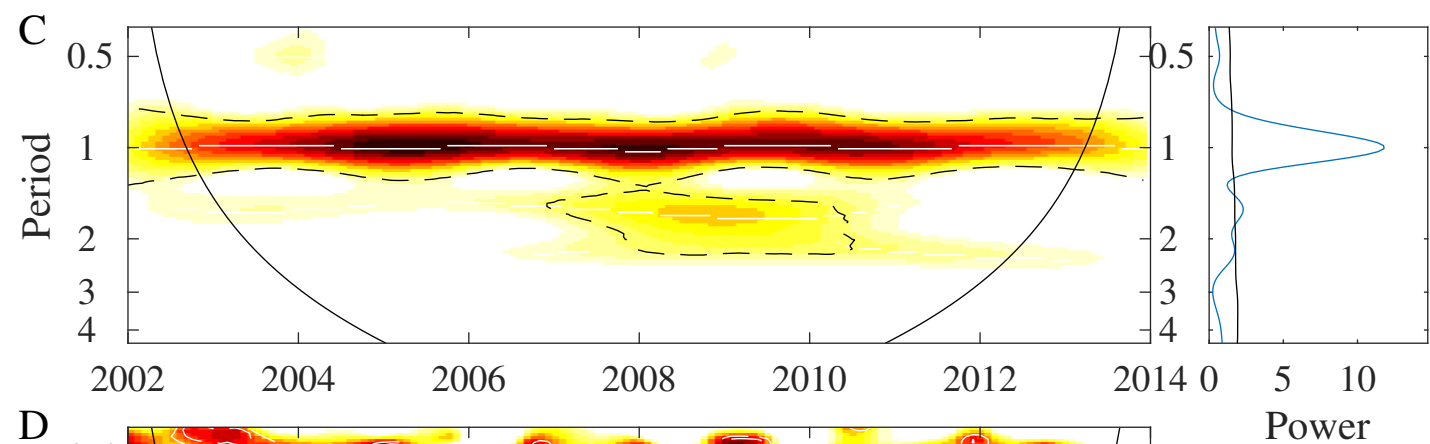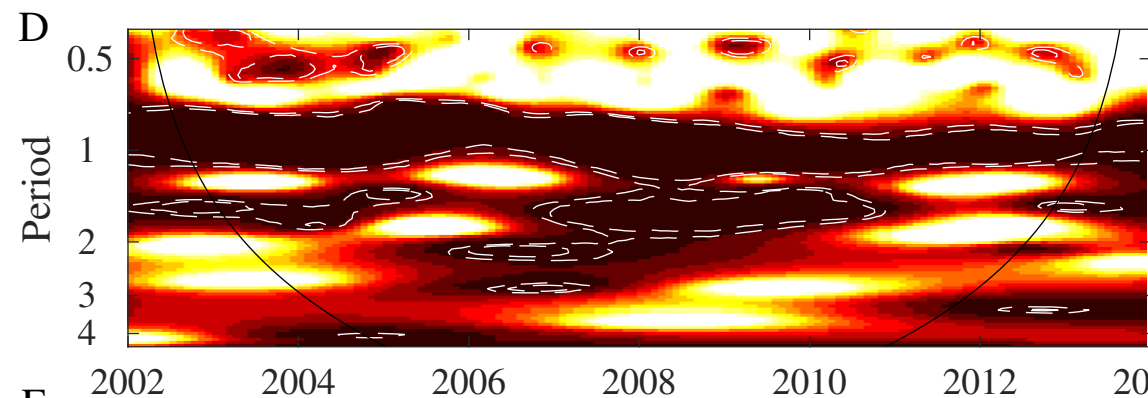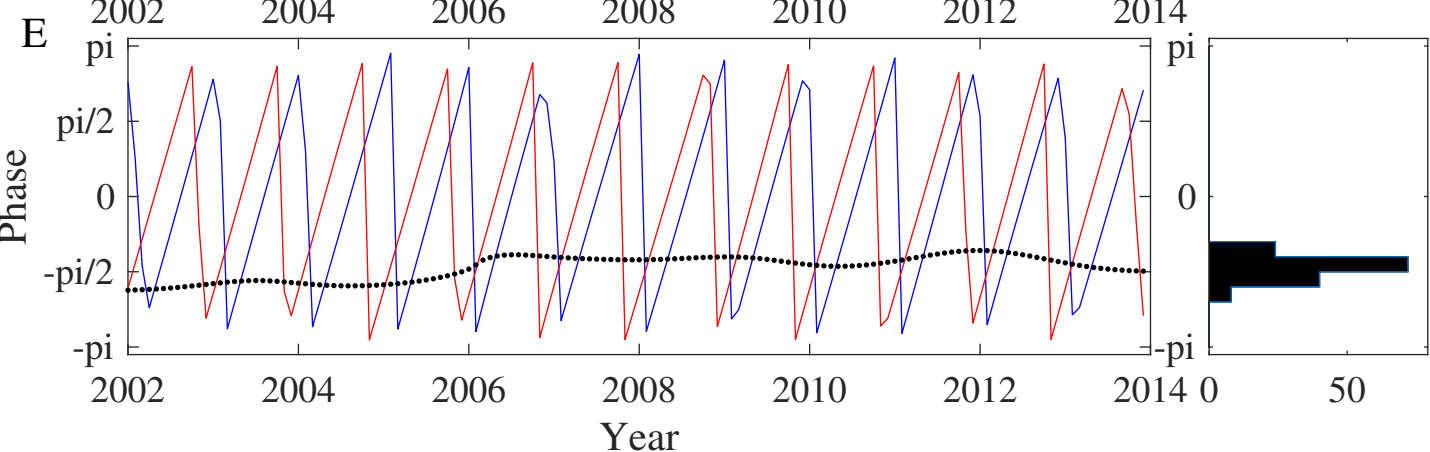

Supplement: S18 Fig — (A) Time evolution of the normalized β(t) (blue line) and normalized average temperature (red line). (B) and (C) Wavelet Power Spectrum (WPS) [48,70] of the two time series. The graph on the right shows the average WPS. (D) Wavelet coherence [48,70] between the reconstructed β(t) and average temperature. In (B), (C) and (D) the colors code for low values in white to high values in dark red. The dashed lines show the 95% CI computed with adapted bootstrappes [71], in (C) the 90% and the 95% CI have been plotted. (E) The evolution of the phase of the two time series computed based on wavelet decomposition for the seasonal mode, blue dashed line for the normalized β(t) red dashed line for the normalized averaged temperature and black dotted line for their phase difference. The graph on the right shows the distribution of the phase differences. (PDF) [file pcbi.1006211.s021.pdf]

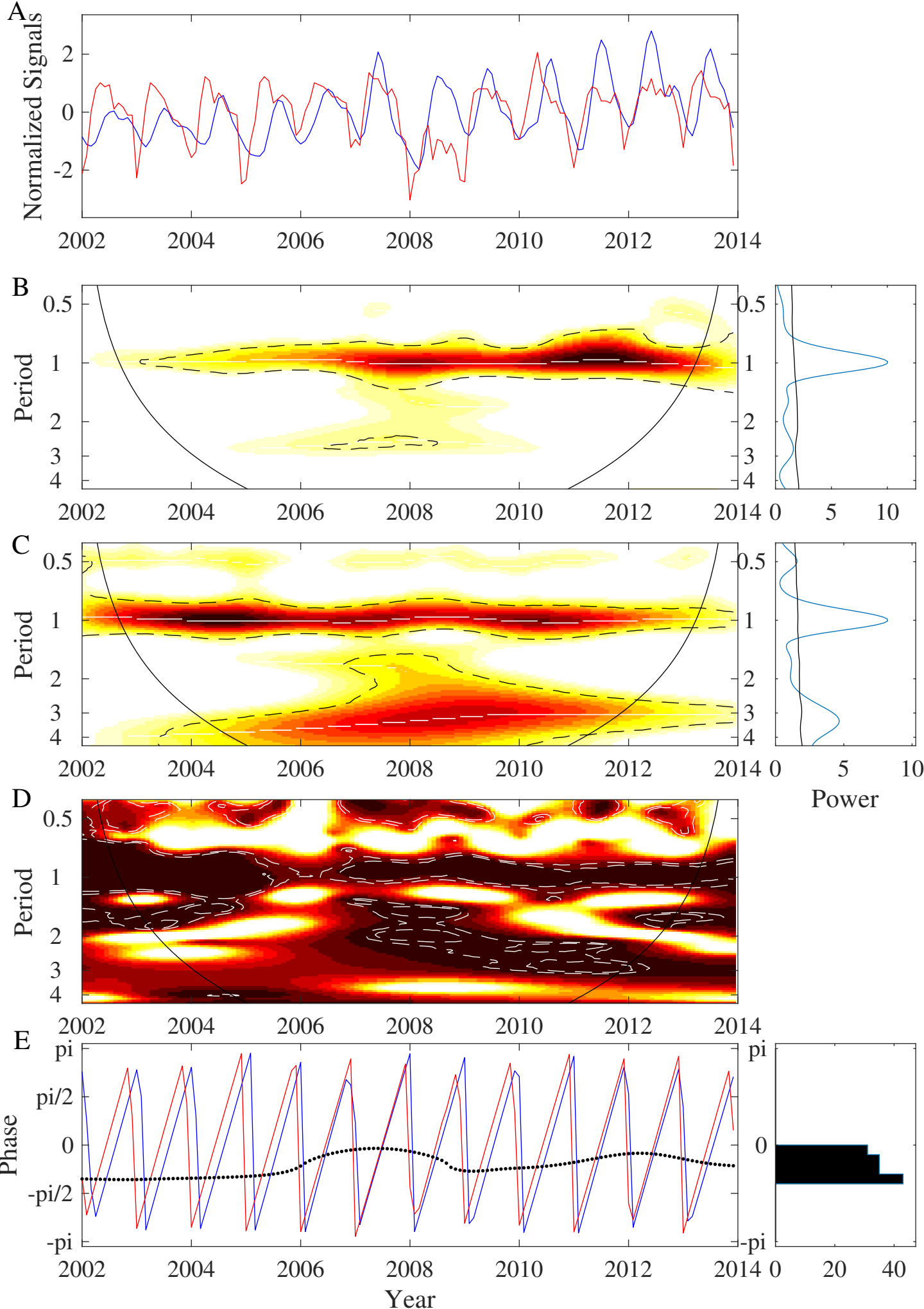

Supplement: S19 Fig — (A) Time evolution of the normalized β(t) (blue line) and normalized average temperature (red line). (B) and (C) Wavelet Power Spectrum (WPS) [48,70] of the two time series. The graph on the right shows the average WPS. (D) Wavelet coherence [48,70] between the reconstructed β(t) and average temperature. In (B), (C) and (D) the colors code for low values in white to high values in dark red. The dashed lines show the 95% CI computed with adapted bootstrappes [71], in (C) the 90% and the 95% CI have been plotted. (E) The evolution of the phase of the two time series computed based on wavelet decomposition for the seasonal mode, blue dashed line for the normalized β(t) red dashed line for the normalized averaged temperature and black dotted line for their phase difference. The graph on the right shows the distribution of the phase differences. (PDF) [file pcbi.1006211.s022.pdf]

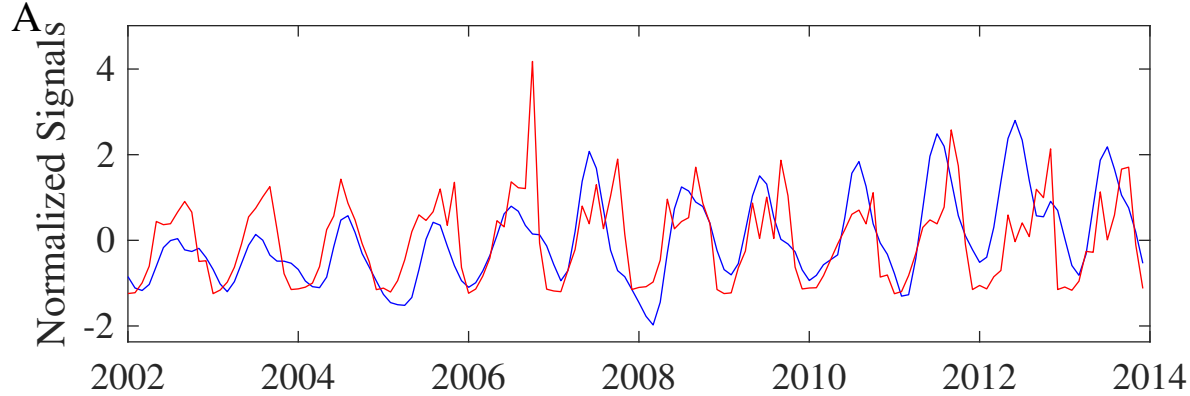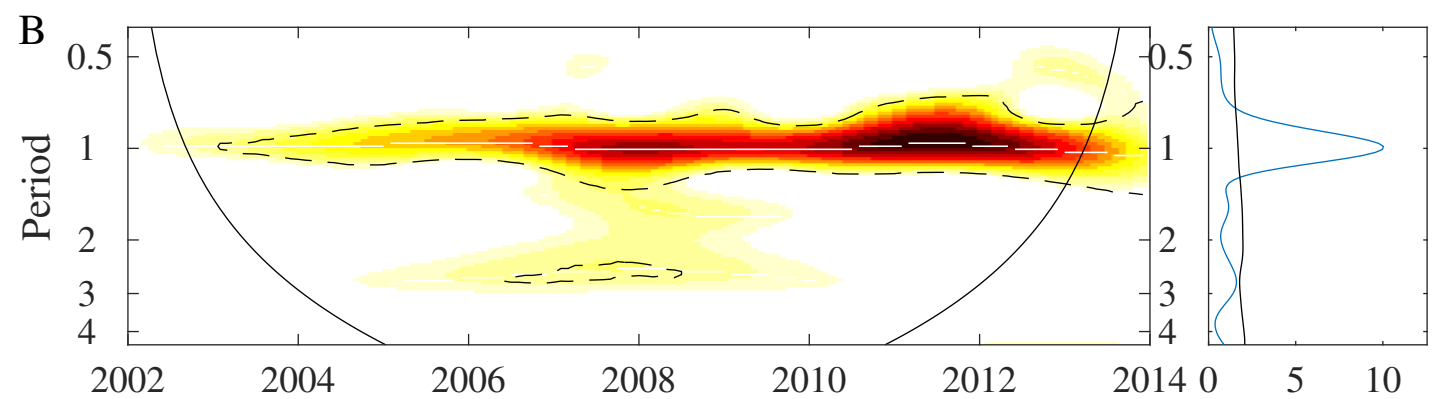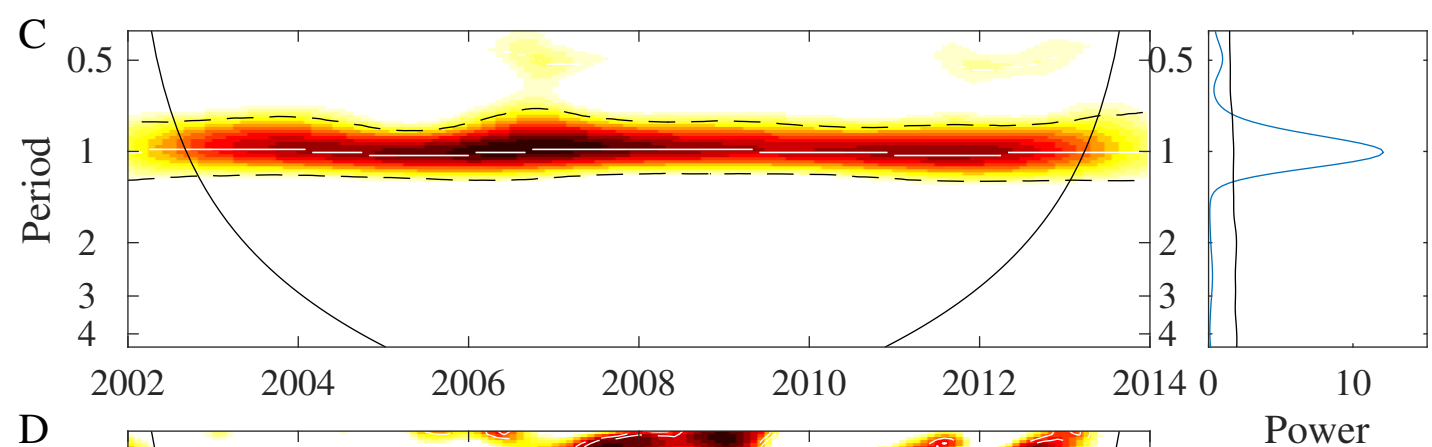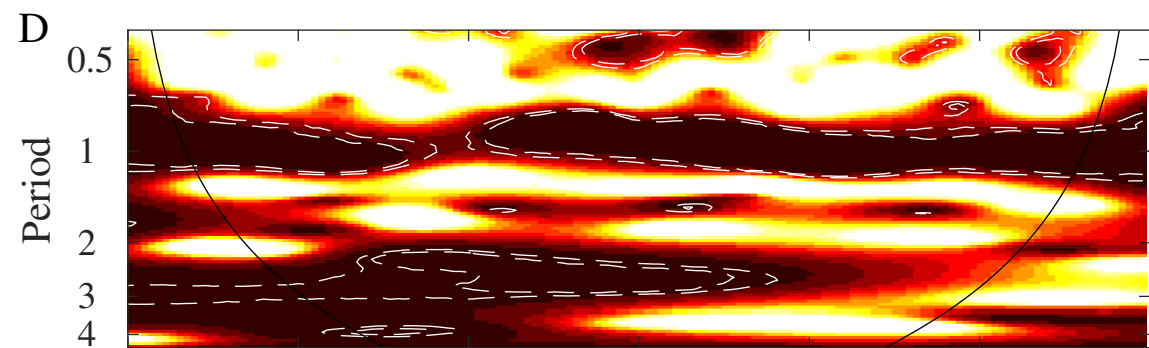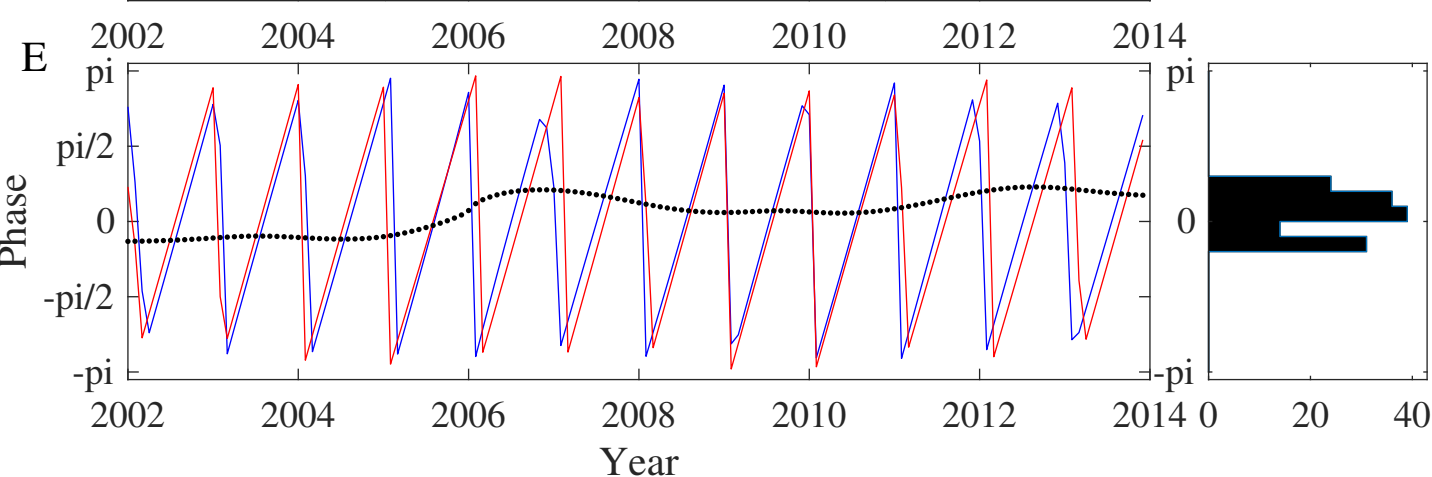

Supplement: S20 Fig — (A) Time evolution of the normalized β(t) (blue line) and normalized monthly rainfall (red line). (B) and (C) Wavelet Power Spectrum (WPS) [48,70] of the two time series. The graph on the right shows the average WPS. (D) Wavelet coherence [48,70] between the reconstructed β(t) and monthly rainfall. In (B), (C) and (D) the colors code for low values in white to high values in dark red. The dashed lines show the 95% CI computed with adapted bootstrappes [71], in (C) the 90% and the 95% CI have been plotted. (E) The evolution of the phase of the two time series computed based on wavelet decomposition for the seasonal mode, blue dashed line for the normalized β(t) red dashed line for the normalized monthly rainfall and black dotted line for their phase difference. The graph on the right shows the distribution of the phase differences. (PDF) [file pcbi.1006211.s023.pdf]

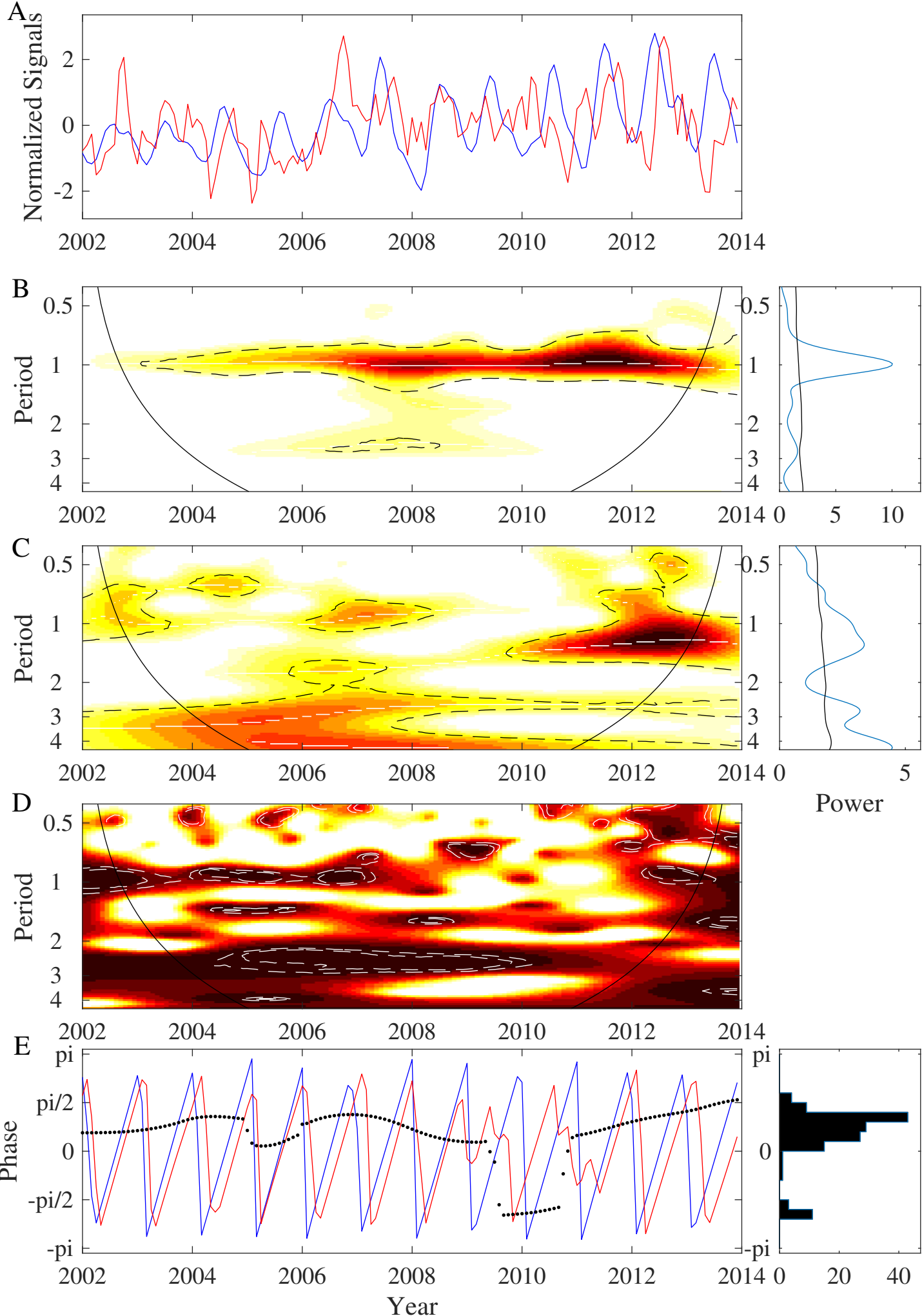

Supplement: S21 Fig — (A) Time evolution of the normalized β(t) (blue line) and normalized DMI (red line). (B) and (C) Wavelet Power Spectrum (WPS) [48,70] of the two time series. The graph on the right shows the average WPS. (D) Wavelet coherence [48,70] between the reconstructed β(t) and DMI. In (B), (C) and (D) the colors code for low values in white to high values in dark red. The dashed lines show the 95% CI computed with adapted bootstrappes [71], in (C) the 90% and the 95% CI have been plotted. (E) The evolution of the phase of the two time series computed based on wavelet decomposition for the seasonal mode, blue dashed line for the normalized β(t) red dashed line for the normalized DMI and black dotted line for their phase difference. The graph on the right shows the distribution of the phase differences. (PDF) [file pcbi.1006211.s024.pdf]
